# 12-year evolution of multimorbidity patterns among older adults based on Hidden Markov Models

**DOI:** 10.18632/aging.204395

**Published:** 2022-11-23

**Authors:** Albert Roso-Llorach, Davide L. Vetrano, Caterina Trevisan, Sergio Fernández, Marina Guisado-Clavero, Lucía A. Carrasco-Ribelles, Laura Fratiglioni, Concepción Violán, Amaia Calderón-Larrañaga

**Affiliations:** 1 Fundació Institut Universitari per a la recerca a l’Atenció Primària de Salut Jordi Gol i Gurina (IDIAPJGol), Barcelona, Spain; 2 Universitat Autònoma de Barcelona, Bellaterra (Cerdanyola de Vallès), Spain; 3 Programa de Doctorat en Metodologia de la Recerca Biomèdica i Salut Pública, Universitat Autònoma de Barcelona, Bellaterra (Cerdanyola del Vallès), Spain; 4 Aging Research Center, Department of Neurobiology, Care Sciences and Society, Karolinska Institutet and Stockholm University, Stockholm, Sweden; 5 Stockholm Gerontology Research Center, Stockholm, Sweden; 6 Department of Medical Sciences, University of Ferrara, Ferrara, Italy; 7 Unidad Docente Multiprofesional de Atención Familiar y Comunitaria Norte, Gerencia Asistencial Atención Primaria, Madrid Health Service, Madrid, Spain; 8 Signal Theory and Communications Department, Universitat Politecnica de Catalunya, Barcelona, Spain; 9 Unitat de Suport a la Recerca Metropolitana Nord, Fundació Institut Universitaria per a la recerca a l’Atenció Primària de Salut Jordi Gol i Gurina (IDIAPJGol), Mataró, Barcelona, Spain

**Keywords:** multimorbidity, older adults, longitudinal population-based study, aging, Hidden Markov Models

## Abstract

Background: The evolution of multimorbidity patterns during aging is still an under-researched area. We lack evidence concerning the time spent by older adults within one same multimorbidity pattern, and their transitional probability across different patterns when further chronic diseases arise. The aim of this study is to fill this gap by exploring multimorbidity patterns across decades of age in older adults, and longitudinal dynamics among these patterns.

Methods: Longitudinal study based on the Swedish National study on Aging and Care in Kungsholmen (SNAC-K) on adults ≥60 years (N=3,363). Hidden Markov Models were applied to model the temporal evolution of both multimorbidity patterns and individuals' transitions over a 12-year follow-up.

Findings: Within the study population (mean age 76.1 years, 66.6% female), 87.2% had ≥2 chronic conditions at baseline. Four longitudinal multimorbidity patterns were identified for each decade. Individuals in all decades showed the shortest permanence time in an *Unspecific* pattern lacking any overrepresented diseases (range: 4.6-10.9 years), but the pattern with the longest permanence time varied by age. Sexagenarians remained longest in the *Psychiatric-endocrine and sensorial* pattern (15.4 years); septuagenarians in the *Neuro-vascular and skin-sensorial* pattern (11.0 years); and octogenarians and beyond in the *Neuro-sensorial* pattern (8.9 years). Transition probabilities varied across decades, sexagenarians showing the highest levels of stability.

Interpretation: Our findings highlight the dynamism and heterogeneity underlying multimorbidity by quantifying the varying permanence times and transition probabilities across patterns in different decades. With increasing age, older adults experience decreasing stability and progressively shorter permanence time within one same multimorbidity pattern.

## INTRODUCTION

Extended human longevity is a goal achieved in the last century, and a reality in middle- and high-income countries [1]. Improvements in health resources and medical sciences, and decreases in preventable mortality have been key to living longer [2]. However, increasing life expectancy comes along with a higher burden of chronic diseases [3]. The coexistence of multiple chronic diseases in a single person is known as multimorbidity. Multimorbidity is associated with a higher risk of polypharmacy and decreased quality of life, and challenges the decision-making of clinicians that lack effective guidelines for the management and treatment of patients with cohexisting complex diseases [4].

In an attempt to understand how chronic diseases are inter-related, several studies have explored so-called multimorbidity patterns [5–7]. In a previous systematic review, three patterns of multimorbidity involving cardiometabolic diseases, mental health problems, and musculoskeletal disorders have been consistently suggested to be the most prevalent in the older population [5]. Diseases cluster in specific patterns due to common pathophysiological pathways and risk factors, or because they may be the cause or consequence of other coexisting diseases. Along with the above mentioned patterns, a high number of less reproducible and sparse disease combinations have been described, often inconsistently across studies. Several factors may explain such disparate observations: first, the use of cross-sectional designs, which do not account for the dynamic nature of multimorbidity in old age; second, the use of different disease lists, spanning from less than ten to more than two hundred conditions; and third, the employement of statistical methods that cannot properly manage the complexity of the phenomenon. Recently, several advanced machine-learning techniques such as non-hierarchical and hierarchical clustering tehcniques have been used to explore multimorbidity patterns.

Exploring how multimorbidity patterns evolve throughout people’s lives and the time subjects remain within specific patterns is still an under-researched area [7, 8]. The understanding of how diseases cluster longitudinally in specific age groups would pave the way to the design of new prognostic tools, as well as new preventive and, eventually, therapeutic approaches. Hidden Markov Models (HMM) overcome several of the limitations of previously employed methods, which were unable to account for the variability in chronic disease interactions throughout time [9]. HMM consider diseases in each person to be random variables conditioned by a hidden state or cluster. Despite the technique’s potential, only one previous register-based study has used HMM for the longitudinal study of multimorbidity [9], but the folllow-up time was insufficient to draw any relevant conclusions. Cohort studies with homogeneously collected data over long periods of time represent a unique resource for the longitudinal analysis of multimorbidity patterns, and their use for such a purpose is warranted.

The aims of this study were: 1) to explore longitudinal multimorbidity patterns across decades of age after 60 using HMM, and 2) to detect the dynamics underlying such patterns in terms of the time subjects remained within the same pattern, and the probability of transitioning across different patterns.

## RESULTS

### Multimorbidity patterns

The study population included 3,363 individuals aged 60+ of whom 87.2% had multimorbidity at baseline. Participants’ mean age at baseline was 76.1 years, and 66.6% were female. Over the 12-year follow-up, 1346 (40%) deaths occurred (25% within the first 6 years and 15% within the next 6 years). Moreover, 719 (21.4%) individuals dropped out (13.7% within the first 6 years and 7.7% within the next 6 years). Descriptive statistics of each age cohort at each follow-up wave can be found in Table 1.

**Table 1:** Sociodemographic, clinical, and functional characteristics of the study population by baseline age group (N=3,363).

	***Sexagenarians***			***Septuagenarians***			***Octogenarians and beyond***		
	**Baseline N=1304**	**6 years follow-up N=1045**	**12 years follow-up N=846**	**Baseline N=939**	**6 years follow-up N=639**	**12 years follow-up N=358**	**Baseline N=1120**	**6 years follow-up N=374**	**12 years follow-up N=94**
Age, mean (SD)	63.0 (2.91)	68.9 (2.89)	74.9 (2.88)	75.3 (3.00)	81.1 (2.98)	86.6 (2.89)	87.9 (5.10)	91.5 (4.11)	95.5 (2.84)
Female, n (%)	735 (56.4%)	603 (57.7%)	503 (59.5%)	598 (63.7%)	419 (65.6%)	245 (68.4%)	849 (75.8%)	276 (73.8%)	71 (75.5%)
Education, n (%)									
Elementary	93 (7.14%)	61 (5.84%)	45 (5.32%)	150 (16.1%)	95 (14.9%)	48 (13.4%)	347 (31.7%)	95 (25.7%)	22 (23.4%)
High school	561 (43.1%)	445 (42.6%)	346 (40.9%)	514 (55.1%)	343 (53.7%)	189 (52.8%)	576 (52.6%)	197 (53.4%)	53 (56.4%)
University	648 (49.8%)	539 (51.6%)	455 (53.8%)	269 (28.8%)	201 (31.5%)	121 (33.8%)	173 (15.8%)	77 (20.9%)	19 (20.2%)
# chronic diseases, mean (SD)	2.72 (1.78)	4.87 (2.78)	7.70 (3.57)	4.24 (2.28)	7.71 (3.46)	12.0 (4.56)	5.47 (2.51)	9.70 (3.58)	14.2 (4.41)
# drugs, mean (SD)	2.66 (2.77)	4.13 (3.37)	5.18 (3.92)	4.39 (3.42)	6.10 (3.92)	7.44 (4.50)	5.37 (3.48)	7.25 (3.97)	8.47 (4.46)
Walking speed, mean (SD)	1.26 (0.31)	1.20 (0.35)	1.08 (0.35)	1.00 (0.38)	0.79 (0.41)	0.66 (0.41)	0.54 (0.41)	0.43 (0.36)	0.37 (0.35)
MMSE, mean (SD)	29.3 (1.45)	28.7 (1.59)	28.5 (2.27)	28.4 (3.32)	26.9 (4.25)	25.5 (5.73)	24.8 (7.39)	24.1 (6.99)	21.9 (8.64)

Abbreviations: MMSE, Mini Mental State Examination; SD, standard deviation.

In the three age groups, a total of 44, 49 and 47 chronic disease categories, respectively, showed a median prevalence ≥2% during the study period, and were thus included in the HMM estimations (Supplementary Table 1). Overall, four multimorbidity patterns were identified for each age group, and two additional patterns were artificially added to account for death and dropout during the follow-up period (Supplementary Table 2).

Among sexagenarians, subjects in the *Unspecific* pattern were the youngest across all follow-ups, while those in the *Cardiovascular and anemia* pattern were the oldest (Supplementary Table 3). Subjects in the *Cardio-metabolic* pattern were more frequently male while those in the *Psychiatric-endocrine and sensorial* pattern were more likely to be female. Subjects in the latter pattern showed the highest level of education.

Among septuagenarians, subjects in the *Unspecific* pattern were the youngest, while those in the *Neuro-vascular and skin-sensorial* pattern were the oldest. Subjects in the *Cardiovascular and diabetes* pattern were more frequently male while those in the *Neuro-vascular and skin-sensorial* and *Neuro-psychiatric and sensorial* patterns were more likely to be female. Subjects in the *Cardiovascular and diabetes* pattern had the lowest proportion of university education.

In the group of octogenarians and beyond, those in the *Respiratory-circulatory and skin* pattern were the youngest, while those in the *Cardio-respiratory and neurological* were the oldest. All patterns had a higher proportion of females. Subjects in the *Neuro-sensorial* pattern showed the highest level of education.

### Evolution and transitions across multimorbidity patterns

**Figure 1:**
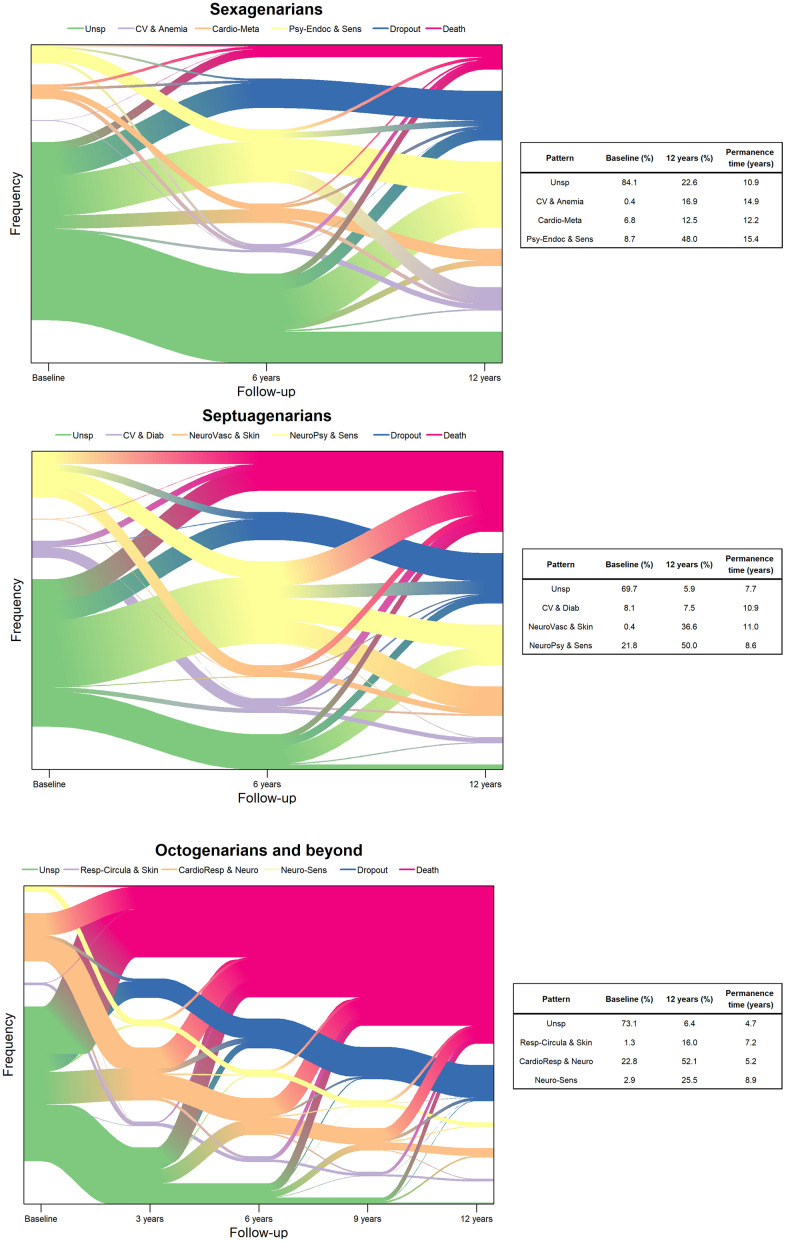
**Evolution and transitions of multimorbidity patterns over time by age group (N=3,363).** Sexagenarians: Unspecific (Unsp); Cardiovascular and anemia (CV and Anemia); Cardio-metabolic (Cardio-Meta) and Psychiatric-endocrine and sensorial (Psy-Endoc and Sens). Septuagenarians: Unspecific (Unsp); Cardiovascular and diabetes (CV and Diab); Neuro-vascular and skin-sensorial (NeuroVasc and Skin); and Neuro-psychiatric and sensorial (NeuroPsy and Sens). Octogenarians and beyond: Unspecific (Unsp); Respiratory-circulatory and skin (Resp-Circula and Skin); Cardio-respiratory and neurological (CardioResp and Neuro); and Neuro-sensorial (Neuro-Sens).

The evolution and transitions of and among multimorbidity patterns are graphically represented through river plots in Figure 1. For all age groups, pattern prevalence varied over time, showing that people commonly transition from one pattern to another. A general trend was that the most represented patterns at baseline (i.e., containing the healthiest subjects) evolved towards smaller ones over time, and the smallest ones (i.e., presumably containing the sickest subjects) tended to become larger over time. For example, among sexagenarians, subjects in the *Unspecific* pattern represented 80% of the study population at baseline, but the figure went down to 52.4% after 6 years and to 22.6% after 12 years. The prevalence of the death and dropout patterns increased in older age groups; an important part of the transitions among octogenarians and beyond were in fact towards death.

The estimated mean permanence times were computed for each age group. As an example, for sexagenarians belonging to the *Cardiovascular and anemia* pattern at baseline, it was estimated that they would remain in the same pattern for a mean time of 14.9 years before transitioning to other patterns. In all age groups, the *Unspecific* patterns showed the shortest sojourn times, and the *Psychiatric-endocrine and sensorial, Neuro-vascular and skin-sensorial* and *Neuro-sensorial* were the patterns with the longest sojourn time for sexagenarians, septuagenarians and octogenarians and beyond, respectively.

**Figure 2:**
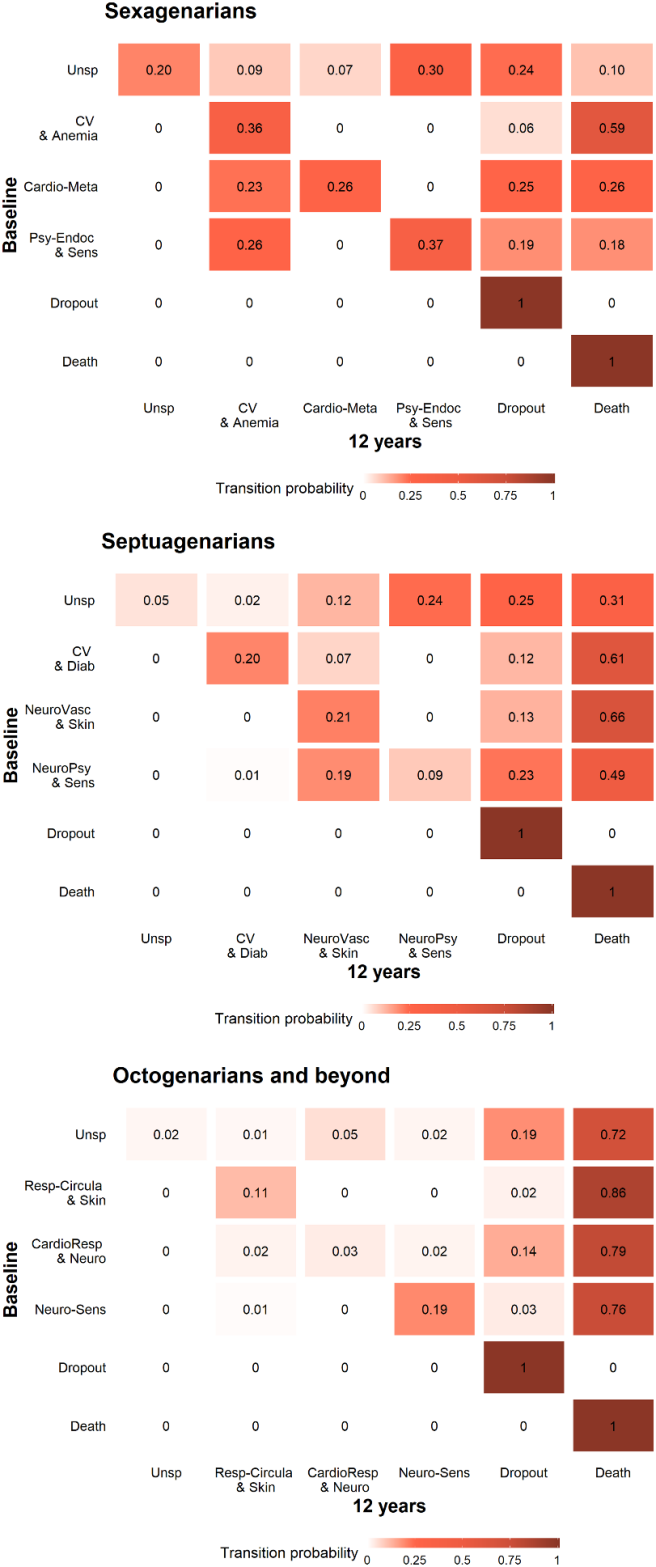
**Transition probability matrices by age group from baseline to the 12-year follow-up (N=3,363).** Sexagenarians: Unspecific (Unsp); Cardiovascular and anemia (CV and Anemia); Cardio-metabolic (Cardio-Meta) and Psychiatric-endocrine and sensorial (Psy-Endoc and Sens). Septuagenarians: Unspecific (Unsp); Cardiovascular and diabetes (CV and Diab); Neuro-vascular and skin-sensorial (NeuroVasc and Skin); and Neuro-psychiatric and sensorial (NeuroPsy and Sens). Octogenarians and beyond: Unspecific (Unsp); Respiratory-circulatory and skin (Resp-Circula and Skin); Cardio-respiratory and neurological (CardioResp and Neuro); and Neuro-sensorial (Neuro-Sens).

The transition probability matrices by age group are shown in Figure 2. Regarding the interpretation of these probabilities, the models show that, for example, sexagenarians belonging to the *Unspecific* pattern at baseline had a probability of 0.9% of transitioning to the *Cardiovascular and anemia* pattern and of 20.0% of staying in the same pattern in the next 12 years. In general, sexagenarians showed the highest levels of stability, as the probabilities of staying in the same pattern were higher than in the other age groups. More specifically, among sexagenarians, the most likely transition between patterns was from the *Unspecific* to the *Psychiatric-endocrine and sensorial* pattern (30.0%) after 12 years. Among septuagenarians, the most likely transition was from the *Unspecific* to the *Neuro-psychiatric and sensorial* pattern (24.0%) after 12 years. Finally, in octogenarians and beyond, the transition from the *Unspecific* to the *Cardio-respiratory and neurological* pattern (5.0%) after 12 years was the likeliest. The *Cardiovascular and anemia*, *Neuro-vascular and skin-sensorial*, and *Respiratory-circulatory and skin* patterns showed the highest probabilities of transitioning to death after 12 years in the three age groups, respectively.

### Characterization of multimorbidity patterns

**Figure 3:**
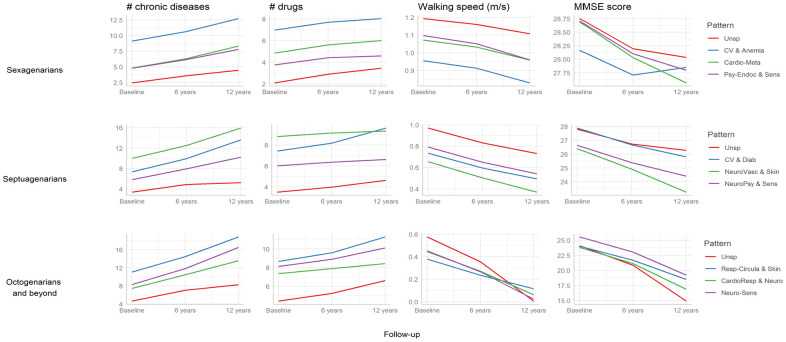
**Longitudinal trends (predicted values from linear mixed models) in clinical and functional characteristics associated with the multimorbidity patterns by age group (N=3,363).** Sexagenarians: Unspecific (Unsp); Cardiovascular and anemia (CV and Anemia); Cardio-metabolic (Cardio-Meta) and Psychiatric-endocrine and sensorial (Psy-Endoc and Sens). Septuagenarians: Unspecific (Unsp); Cardiovascular and diabetes (CV and Diab); Neuro-vascular and skin-sensorial (NeuroVasc and Skin); and Neuro-psychiatric and sensorial (NeuroPsy and Sens). Octogenarians and beyond: Unspecific (Unsp); Respiratory-circulatory and skin (Resp-Circula and Skin); Cardio-respiratory and neurological (CardioResp and Neuro); and Neuro-sensorial (Neuro-Sens). MMSE: Mini Mental State Examination.

Estimations of the longitudinal trends (predicted values from linear mixed models) for different clinical and functional variables by patterns and for each age group are shown in Figure 3. An increasing trend was observed for the number of chronic conditions and drugs across age groups, with subjects in the *Unspecific* patterns consistently showing the lowest values. Conversely, a decreasing trend was observed for walking speed and MMSE in all age groups. While subjects in the *Unspecific* patterns showed the slowest changes over time, except for octogenarians, those in the patterns characterized by cardiovascular and/or neurological diseases showed the worse baseline values and fastest declines for all studied variables.

## DISCUSSION

In this study we identified and characterized longitudinal multimorbidity patterns among older adults from a Swedish urban population, and estimated the time they spent in each pattern as well as the probability of transitioning across different patterns throughout a 12-year follow-up period.

Our findings highlight the dynamism and heterogeneity underlying multimorbidity. The dynamism among multimorbidity patterns was reflected by the varying sojourn times across patterns, which differed by age group, and the specific patterns people presented with. In sexagenarians, the average time was 13.3 years, while in octogenarians and beyond, it was 6.5 years. This observation implies that, as expected, the time of permanence in each pattern is greater in the younger age groups, especially when less burdensome patterns are at play. For example, the *Unspecific* pattern was characterized in all age groups by a lack of overrepresentation of any of the low-severity chronic conditions the pattern was composed of (e.g., cardiovascular risk factors, osteoarthritis, hearing impairment, etc.). Consequently, people belonging to this pattern could be regarded as being the healthiest, and thus the target for primary and secondary preventive strategies. Indeed, almost one third of sexagenarians in the *Unspecific* pattern at baseline transitioned to the *Psychiatric and sensorial* pattern, and almost one in ten to the *Cardio-metabolic* pattern during the follow-up. The heterogeneity of multimorbidity was evidenced by the different patterns obtained within, but especially, across age groups. Despite being similar, patterns at different ages represent different states of the disease severity continuum. These different stages may be associated with differential probabilities of developing complications and functional decline, and may trigger different pharmacological and non-pharmacological treatments. In relation to mortality, trajectories characterized by cardiovascular and circulatory diseases were found to concentrate the highest death probabilities. All these aspects may contribute to increase the heterogeneity of the multimorbidity landscape.

Moreover, our study serves as an example of how longitudinal data may be used to explore the trajectories of multimorbidity – that is, the evolution of and transitions among patterns of diseases. To date, studies on patterns of multimorbidity have predominantly focused on analyzing the association between diseases, paying less attention to individuals’ “journeys” in and out of these patterns [5, 10]. This is mainly because most studies, even those using longitudinal data [11], were based on cross-sectional designs. Indeed, studies incorporating the entire longitudinal structure of the data are scarce [12–14]. Studying patterns of multimorbidity longitudinally is a challenging endeavor given that the heterogeneity in disease clustering originates both from the cross-sectional and longitudinal axes. Therefore, to understand the interdependence among diseases when looking at longitudinal multimorbidity patterns, dynamic machine learning methodologies such as the HMM are required. These models integrate a dynamic Bayesian network that accounts for the temporal sequence of the person-level data observed. This allows considering the longitudinal structure of the data (i.e., time series) and the correlations among observations.

Comparing our results with those from previous studies is difficult for the reasons mentioned above. Nevertheless, two previous studies analyzed disease progression and multimorbidity pattern trajectories using primary care electronic health records in the United Kingdom [15] and the Netherlands [16]. The studies by Strauss et al. [15] and Lappenschaar et al. [16] were carried out on adult populations, older than 35 and 50, and with a follow-up period of 3 and 5 years, respectively; and both included a lower number of chronic diseases than that used in this study. In terms of the analytical approach, the latent class growth models employed by Strauss et al. are designed to identify longitudinal trajectories, but one cannot infer transitions among classes. Also, Lappenschaar et al. used multilevel temporal Bayesian networks, which are aimed at analyzing the relationships between diseases (i.e., networks) but not the transitions across clusters. Other studies [17, 18] have focused on the incidence of new chronic diseases across time, but failed to examine patterns of multimorbidity. In brief, none of the previously applied statistical methods makes it possible to study the evolution and transitions between patterns of multimorbidity. In contrast, when applying HMM, one can explore the variability of chronic disease evolution over time by considering each subject’s diseases as random variables conditioned by a hidden or conglomerate state, which further enables depicting people’s transitions among different patterns of multimorbidity. Other studies looking at multimorbidity patterns within large databases have considered disease trajectories rather than individual trajectories as the main axis of interest [19]. This approach, which is somewhat disease- rather than person-oriented, is limited by the inability to identify homogenous groups of patients. Another example is the work by Giannoula et al., which focused on the identification of complex time-dependent disease associations using dynamic time warping, a machine learning technique [20]. Similar problems are present in the study by Xu et al., which moreover only considered three pathologies [21].

This study has several strengths. First, thanks to the exhaustive clinical evaluation that SNAC-K participants undergo in each follow-up wave, the reliability of the diagnostic data, which moreover integrates data from electronic health records, lab tests and drug use, is optimal. Second, the statistical methods applied allowed us to cluster people by their co-occurring diseases taking both the cross-sectional and longitudinal axes into account: HMM and the fuzzy c-means cluster algorithm. The latter is the choice method for pattern recognition when clusters tend to overlap, which is often the case as older adults show a high prevalence of co-occurring conditions. Furthermore, in this study we were able to explore longitudinal multimorbidity patterns by age group and the time that people remained in each pattern. As far as we know, these aspects have not been previously studied and are key to personalized clinical decision-making. Moreover, by stratifying our study sample by decade age groups, we were able to account for the selection bias inherent to aging cohorts, whereby the oldest age groups tend to represent healthier individuals characterized by better biological and environmental living conditions.

Some limitations must also be considered. First, the relatively small size of the SNAC-K cohort and the further stratification of the study sample into three different age groups led to some of the patterns including few people (i.e., <14 people). However, the methods applied have been shown to be responsive enough for the identification of subgroups of people even in small samples. Additionally, the iterative estimation process and the number of realizations allowed us to maximize the likelihood of the models applied given the data. Second, participant dropout (14% within the first 6 years and 8% within the next 6 years) may have affected the cluster definition process. Still, to the best of our knowledge, this is an exceptionally low figure compared with studies of this type. Third, the discontinuous follow-up carried out in SNAC-K (i.e., every 3 or 6 years depending on the age of participants) may have affected the rate of disease detection and, consequently, the longitudinal cluster analysis, especially among people who died or dropped out during the observation period. To adapt to the assumptions of our study design, participant data were analyzed in accordance with the available follow-up waves, avoiding any data extrapolation. Last, differences in the baseline composition and evolution of patterns across age groups could be due to variations in exposure history, and not only to age, given that there is up to 40 years of a gap between the youngest and oldest subjects at study baseline.

The analysis of longitudinal multimorbidity patterns is fundamental for the provision of personalized medical care that is not based merely on the application of guidelines targeting each chronic condition individually. While some of our findings can be explained through known pathophysiological mechanisms, others may serve to generate new hypotheses worth exploring in future studies. Our statistical approach enabled us to model the evolution and transitions of multimorbidity over time, and the results of this could be applied in the interests of healthier aging. Moreover, the age-stratified analyses allowed us to identify which disease combinations and transitions were more prevalent in each decade. This information is key to defining specific care plans to prevent or delay the negative consequences of the most frequent diseases identified. The characterization of multimorbidity patterns using HMM could moreover be expanded, for instance, by aggregating information on complementary health indicators such as frailty and biological and physiological variables, which could further optimize patient stratification and management efforts.

Our study provides evidence that multimorbidity is dynamic and heterogeneous in old age. With increasing age, older adults experience decreasing clinical stability and progressively shorter permanence time within one same multimorbidity pattern. Moreover, a significant proportion ranging between 5.9%-22.6% belongs to an *Unspecific* pattern with a low burden of diseases and a promising preventive potential. Adding new variables related to drug use, environmental and genetic factors, and/or frailty to the longitudinal analysis of multimorbidity patterns may allow optimizing the epidemiological understanding and applicability of these models for patient-tailored prevention and management strategies.

## MATERIALS AND METHODS

### Study population

Longitudinal data from the population-based Swedish National study on Aging and Care in Kungsholmen (SNAC-K) was used [22]. The study population consisted of adults ≥60 years of age living in the community or in institutions in the Kungsholmen district of Stockholm, Sweden. A random sample of 11 age cohorts (ages 60, 66, 72, 78, 81, 84, 87, 90, 93, 96 and ≥99) born between 1898 and 1943 (the youngest and oldest age cohorts were oversampled) was invited to participate in the study. People who agreed to participate were evaluated for the first time between 2001 and 2004. Participants who were <78 years of age were then followed up every six years and participants ≥78 every three years. The present study is based on data collected at baseline, the six-year follow-up, and the 12-year follow-up. At baseline, 3363 people were examined (participation rate: 73%). For our study, the sample was stratified into three age groups: sexagenarians (age cohorts of 60 and 66 years), septuagenarians (age cohorts of 72 and 78 years) and octogenarians and beyond (age cohorts of 81 years and over).

### Chronic diseases

At each follow-up wave, SNAC-K participants undergo an approximately five-hour-long comprehensive clinical and functional assessment carried out by trained physicians, nurses, and neuropsychologists. Physicians collect information on diagnoses via physical examination, medical history, examination of medical charts, self-reported information, and/or proxy interviews. Clinical parameters, lab tests, drug information, and inpatient and outpatient care data are also used to identify specific conditions. All diagnoses are coded in accordance with the International Classification of Diseases, 10th revision (ICD-10). In the current study we classified all the ICD-10 codes into 60 chronic disease categories in accordance with a clinically driven methodology [23]. In SNAC-K, drugs are coded in accordance with the Anatomical Therapeutic Chemical (ATC) classification.

### Covariates

Information on demographics (age, sex, education) was collected during nurse interviews. We divided education into elementary, secondary, university or higher. Information about vital status was derived from death certificates provided by Statistics Sweden, the Swedish governmental statistics agency. Survival status was assessed throughout the follow-up period. Participants were considered lost to follow-up if they or a proxy declined to participate, could not be contacted, had moved out of the study area, or cancelled an assessment. Walking speed (m/s) was assessed by asking participants to walk 6 m at their usual speed or 2.44 m if the participant reported walking quite slowly [24]. Cognitive status was assessed by physicians using the Mini-Mental State Examination (MMSE), with a score range of 30 at best to 0 at worst [25].

### Statistical analysis

The sample characteristics at baseline, the 6-year follow-up and the 12-year follow-up for all age groups were described as appropriate. Additionally, 3-year and 9-year follow-up data was considered for the group of octogenarians and beyond.

To model the temporal evolution of multimorbidity patterns and individuals’ transitions across these patterns, a dynamic random process represented by a HMM was assumed [9]. Disease information from all individuals and across all follow-up waves is used by the HMM to identify so-called hidden states (i.e., longitudinal multimorbidity pattern). HMM estimates the transition probabilities between patterns, i.e., the probability that any individual moves from one pattern to another in a given time-frame. Furthermore, by using HMM, one can examine individuals’ probability of following different longitudinal multimorbidity patterns, and subsequently identify the one that is most likely to happen.

The dataset was pre-processed by applying a Multiple Correspondence Analysis (MCA) to the categorical features (i.e., diseases), in order to reduce the dimensionality of the longitudinal dataset. To prevent statistical noise and spurious findings from the models, only diseases that achieved a median prevalence of 2% across all follow-up waves were included (Supplementary Table 1). Afterwards, a fuzzy segmentation procedure (Fuzzy C-means algorithm, FCM) [11] was applied on the new dataset to identify an initial set of clusters, which was used to initialize some of the HMM parameters in the next stage. Finally, two more clusters were added in order to account for dropout and/or death.

The set of HMM parameters, composed of the initial cluster probabilities, the inter-cluster transition probabilities and the emission distributions provided by the FCM, were fitted into the observation dataset by applying the Baum-Welch (BW) algorithm. This made it possible to infer the longitudinal trajectories followed by each individual. The best cluster trajectory was identified by maximizing the probability of the observed sequence conditioned to the computed model parameters (Viterbi Algorithm). To validate the model, a comparison between BW and Viterbi transition probability matrices was conducted, showing a good agreement between theoretical and observed values [26].

The time unit considered for each transition across clusters/states was the time between follow-up waves, 6 years for sexagenarians and septuagenarians and 3 years for octogenarians and beyond. The time spent in a specific cluster/state before moving to other clusters/states was assumed to follow a geometric distribution. Subsequently, the expected average time spent or mean sojourn (permanence) time was computed.

To optimize the performance of the selected mathematical model, the iterative process involved in the application of the BW algorithm was initialized using a range of 100 different values of the parameters to be learned. The best model was selected using a procedure that is equivalent to applying the Bayes Information Criterion to choose the best set of HMM parameters [9].

### Multimorbidity patterns

For each age group, a final number of longitudinal patterns was selected. To evaluate the consistency and utility of the final clusters, we contrasted the clinical relevance of our findings in the context of previous literature, and we dicussed the findings within the research team (2 GPs, 2 geriatricians, 3 epidemiologists and 2 statisticians).

To characterize the multimorbidity patterns, we calculated the frequency of chronic diseases in each cluster. Observed/expected ratios (O/E-ratios) were calculated by dividing the prevalence of a given disease within a cluster by its prevalence in the overall population. The exclusivity of different diseases, defined as the fraction of participants with the disease in the cluster over the total number of participants with the disease, was also calculated. We considered a disease to be associated with a given cluster of individuals when the O/E ratio was ≥2 or the exclusivity was ≥ 20% [12]. Such criteria were used to name multimorbidity patterns after the diseases that predominantly characterized them.

The longitudinal trends of clinical and functional characteristics (no. of chronic diseases, no. of drugs, walking speed and MMSE) associated with the multimorbidity patterns were estimated through linear mixed models, assuming a random intercept and including an interaction between the patterns and follow-up time, both as linear and quadratic. The models were additionally adjusted by age, sex and education.

The analyses were carried out using Stata version 17 and R version 4.1.2. The significance level was set at α=0.05.

## Supplementary Material

Supplementary Table 1

Supplementary Table 2

Supplementary Table 3
