# Supplementary material for: 12-year evolution of multimorbidity patterns among older adults based on Hidden Markov Models
Source: Aging (Albany NY). 2022 Nov 23;14(24):9805–17. doi: 10.18632/aging.204395 (PMC9831736; doi:10.18632/aging.204395)
Supplement: Supplementary Table 1 [file aging-14-204395-s001.docx]

**Supplementary Table 1. Disease prevalence by age group and follow-up wave.**

*Sexagenarians*

|  | N_Baseline | Prev_Baseline | N_6 years | Prev_6 years | N_12 years | Prev_12 years | median |
| --- | --- | --- | --- | --- | --- | --- | --- |
| Hypertension | 797 | 61.12 | 765 | 73.21 | 672 | 79.43 | **73.21** |
| Dyslipidemia | 678 | 51.99 | 673 | 64.4 | 600 | 70.92 | **64.4** |
| Osteoarthritis and other degenerative joint diseases | 129 | 9.89 | 297 | 28.42 | 419 | 49.53 | **28.42** |
| Obesity | 202 | 15.49 | 209 | 20 | 189 | 22.34 | **20** |
| Other musculoskeletal and joint diseases | 52 | 3.99 | 167 | 15.98 | 260 | 30.73 | **15.98** |
| Solid neoplasms | 76 | 5.83 | 157 | 15.02 | 225 | 26.6 | **15.02** |
| Colitis and related diseases | 76 | 5.83 | 146 | 13.97 | 200 | 23.64 | **13.97** |
| Cataract and other lens diseases | 17 | 1.3 | 142 | 13.59 | 342 | 40.43 | **13.59** |
| Chronic kidney diseases | 130 | 9.97 | 131 | 12.54 | 140 | 16.55 | **12.54** |
| Depression and mood diseases | 113 | 8.67 | 122 | 11.67 | 126 | 14.89 | **11.67** |
| Diabetes | 91 | 6.98 | 120 | 11.48 | 112 | 13.24 | **11.48** |
| Other eye diseases | 23 | 1.76 | 116 | 11.1 | 222 | 26.24 | **11.1** |
| Dorsopathies | 82 | 6.29 | 115 | 11 | 148 | 17.49 | **11** |
| Thyroid diseases | 103 | 7.9 | 115 | 11 | 109 | 12.88 | **11** |
| Neurotic, stress-related and somatoform diseases | 40 | 3.07 | 94 | 9 | 122 | 14.42 | **9** |
| Ischemic heart disease | 73 | 5.6 | 92 | 8.8 | 108 | 12.77 | **8.8** |
| Asthma | 82 | 6.29 | 90 | 8.61 | 95 | 11.23 | **8.61** |
| Other genitourinary diseases | 17 | 1.3 | 88 | 8.42 | 228 | 26.95 | **8.42** |
| Esophagus, stomach and duodenum diseases | 52 | 3.99 | 85 | 8.13 | 132 | 15.6 | **8.13** |
| Anemia | 45 | 3.45 | 77 | 7.37 | 107 | 12.65 | **7.37** |
| Prostate diseases | 32 | 2.45 | 72 | 6.89 | 112 | 13.24 | **6.89** |
| COPD, emphysema, chronic bronchitis | 41 | 3.14 | 67 | 6.41 | 80 | 9.46 | **6.41** |
| Osteoporosis | 30 | 2.3 | 66 | 6.32 | 97 | 11.47 | **6.32** |
| Autoimmune diseases | 34 | 2.61 | 65 | 6.22 | 80 | 9.46 | **6.22** |
| Deafness, hearing impairment | 24 | 1.84 | 64 | 6.12 | 143 | 16.9 | **6.12** |
| Allergy | 26 | 1.99 | 62 | 5.93 | 91 | 10.76 | **5.93** |
| Inflammatory arthropathies | 41 | 3.14 | 59 | 5.65 | 82 | 9.69 | **5.65** |
| Atrial fibrillation | 36 | 2.76 | 57 | 5.45 | 95 | 11.23 | **5.45** |
| Sleep disorders | 32 | 2.45 | 55 | 5.26 | 71 | 8.39 | **5.26** |
| Cerebrovascular disease | 33 | 2.53 | 54 | 5.17 | 94 | 11.11 | **5.17** |
| Migraine and facial pain syndromes | 38 | 2.91 | 48 | 4.59 | 59 | 6.97 | **4.59** |
| Other cardiovascular diseases | 23 | 1.76 | 48 | 4.59 | 62 | 7.33 | **4.59** |
| Chronic pancreas, biliary tract and gallbladder diseases | 23 | 1.76 | 42 | 4.02 | 45 | 5.32 | **4.02** |
| Ear, nose, throat diseases | 15 | 1.15 | 41 | 3.92 | 100 | 11.82 | **3.92** |
| Glaucoma | 18 | 1.38 | 38 | 3.64 | 56 | 6.62 | **3.64** |
| Other psychiatric and behavioral diseases | 20 | 1.53 | 36 | 3.44 | 50 | 5.91 | **3.44** |
| Heart failure | 17 | 1.3 | 34 | 3.25 | 61 | 7.21 | **3.25** |
| Other metabolic diseases | 18 | 1.38 | 34 | 3.25 | 46 | 5.44 | **3.25** |
| Peripheral neuropathy | 11 | 0.84 | 33 | 3.16 | 76 | 8.98 | **3.16** |
| Venous and lymphatic diseases | 11 | 0.84 | 30 | 2.87 | 62 | 7.33 | **2.87** |
| Other neurological diseases | 18 | 1.38 | 29 | 2.78 | 42 | 4.96 | **2.78** |
| Blindness, visual impairment | 10 | 0.77 | 26 | 2.49 | 41 | 4.85 | **2.49** |
| Peripheral vascular disease | 12 | 0.92 | 26 | 2.49 | 30 | 3.55 | **2.49** |
| Cardiac valve diseases | 9 | 0.69 | 25 | 2.39 | 59 | 6.97 | **2.39** |
| Inflammatory bowel diseases | 14 | 1.07 | 18 | 1.72 | 18 | 2.13 | 1.72 |
| Chronic infectious diseases | 6 | 0.46 | 17 | 1.63 | 19 | 2.25 | 1.63 |
| Other digestive diseases | 9 | 0.69 | 17 | 1.63 | 24 | 2.84 | 1.63 |
| Blood and blood forming organ diseases | 8 | 0.61 | 16 | 1.53 | 20 | 2.36 | 1.53 |
| Other respiratory diseases | 9 | 0.69 | 15 | 1.44 | 15 | 1.77 | 1.44 |
| Parkinson and parkinsonism | 5 | 0.38 | 15 | 1.44 | 21 | 2.48 | 1.44 |
| Dementia | 6 | 0.46 | 12 | 1.15 | 28 | 3.31 | 1.15 |
| Hematological neoplasms | 6 | 0.46 | 12 | 1.15 | 9 | 1.06 | 1.06 |
| Chronic ulcer of the skin | 5 | 0.38 | 11 | 1.05 | 16 | 1.89 | 1.05 |
| Chronic liver diseases | 4 | 0.31 | 11 | 1.05 | 8 | 0.95 | 0.95 |
| Bradycardias and conduction diseases | 5 | 0.38 | 9 | 0.86 | 12 | 1.42 | 0.86 |
| Other skin diseases | 1 | 0.08 | 8 | 0.77 | 21 | 2.48 | 0.77 |
| Epilepsy | 6 | 0.46 | 7 | 0.67 | 9 | 1.06 | 0.67 |
| Schizophrenia and delusional diseases | 6 | 0.46 | 6 | 0.57 | 2 | 0.24 | 0.46 |
| Multiple sclerosis | 2 | 0.15 | 2 | 0.19 | 1 | 0.12 | 0.15 |
| Chromosomal abnormalities | 0 | 0 | 0 | 0 | 0 | 0 | 0 |

*Septuagenarians*

|  | N_Baseline | Prev_Baseline | N_6 years | Prev_6 years | N_12 years | Prev_12 years | median |
| --- | --- | --- | --- | --- | --- | --- | --- |
| Hypertension | 710 | 75.61 | 557 | 87.17 | 331 | 92.46 | **87.17** |
| Dyslipidemia | 477 | 50.8 | 401 | 62.75 | 256 | 71.51 | **62.75** |
| Chronic kidney diseases | 335 | 35.68 | 263 | 41.16 | 186 | 51.96 | **41.16** |
| Osteoarthritis and other degenerative joint diseases | 142 | 15.12 | 251 | 39.28 | 201 | 56.15 | **39.28** |
| Cataract and other lens diseases | 60 | 6.39 | 208 | 32.55 | 222 | 62.01 | **32.55** |
| Colitis and related diseases | 111 | 11.82 | 179 | 28.01 | 167 | 46.65 | **28.01** |
| Solid neoplasms | 115 | 12.25 | 165 | 25.82 | 156 | 43.58 | **25.82** |
| Ischemic heart disease | 160 | 17.04 | 148 | 23.16 | 93 | 25.98 | **23.16** |
| Other eye diseases | 47 | 5.01 | 139 | 21.75 | 169 | 47.21 | **21.75** |
| Anemia | 87 | 9.27 | 131 | 20.5 | 127 | 35.47 | **20.5** |
| Other musculoskeletal and joint diseases | 48 | 5.11 | 128 | 20.03 | 141 | 39.39 | **20.03** |
| Deafness, hearing impairment | 75 | 7.99 | 121 | 18.94 | 165 | 46.09 | **18.94** |
| Atrial fibrillation | 101 | 10.76 | 119 | 18.62 | 96 | 26.82 | **18.62** |
| Obesity | 124 | 13.21 | 115 | 18 | 71 | 19.83 | **18** |
| Heart failure | 83 | 8.84 | 109 | 17.06 | 99 | 27.65 | **17.06** |
| Thyroid diseases | 94 | 10.01 | 104 | 16.28 | 74 | 20.67 | **16.28** |
| Depression and mood diseases | 80 | 8.52 | 102 | 15.96 | 82 | 22.91 | **15.96** |
| Osteoporosis | 75 | 7.99 | 101 | 15.81 | 95 | 26.54 | **15.81** |
| Cerebrovascular disease | 82 | 8.73 | 99 | 15.49 | 90 | 25.14 | **15.49** |
| Dementia | 39 | 4.15 | 94 | 14.71 | 81 | 22.63 | **14.71** |
| Diabetes | 103 | 10.97 | 89 | 13.93 | 66 | 18.44 | **13.93** |
| Dorsopathies | 49 | 5.22 | 85 | 13.3 | 88 | 24.58 | **13.3** |
| Other genitourinary diseases | 31 | 3.3 | 79 | 12.36 | 109 | 30.45 | **12.36** |
| Prostate diseases | 59 | 6.28 | 73 | 11.42 | 47 | 13.13 | **11.42** |
| Glaucoma | 42 | 4.47 | 70 | 10.95 | 63 | 17.6 | **10.95** |
| COPD, emphysema, chronic bronchitis | 58 | 6.18 | 69 | 10.8 | 50 | 13.97 | **10.8** |
| Asthma | 67 | 7.14 | 64 | 10.02 | 47 | 13.13 | **10.02** |
| Neurotic, stress-related and somatoform diseases | 30 | 3.19 | 64 | 10.02 | 78 | 21.79 | **10.02** |
| Autoimmune diseases | 54 | 5.75 | 62 | 9.7 | 55 | 15.36 | **9.7** |
| Esophagus, stomach and duodenum diseases | 38 | 4.05 | 62 | 9.7 | 64 | 17.88 | **9.7** |
| Cardiac valve diseases | 35 | 3.73 | 52 | 8.14 | 58 | 16.2 | **8.14** |
| Other psychiatric and behavioral diseases | 18 | 1.92 | 52 | 8.14 | 40 | 11.17 | **8.14** |
| Inflammatory arthropathies | 40 | 4.26 | 51 | 7.98 | 44 | 12.29 | **7.98** |
| Other cardiovascular diseases | 33 | 3.51 | 51 | 7.98 | 55 | 15.36 | **7.98** |
| Blindness, visual impairment | 16 | 1.7 | 45 | 7.04 | 86 | 24.02 | **7.04** |
| Peripheral neuropathy | 18 | 1.92 | 36 | 5.63 | 45 | 12.57 | **5.63** |
| Bradycardias and conduction diseases | 21 | 2.24 | 35 | 5.48 | 25 | 6.98 | **5.48** |
| Other neurological diseases | 27 | 2.88 | 34 | 5.32 | 38 | 10.61 | **5.32** |
| Ear, nose, throat diseases | 9 | 0.96 | 31 | 4.85 | 51 | 14.25 | **4.85** |
| Peripheral vascular disease | 16 | 1.7 | 31 | 4.85 | 21 | 5.87 | **4.85** |
| Sleep disorders | 19 | 2.02 | 30 | 4.69 | 19 | 5.31 | **4.69** |
| Other metabolic diseases | 12 | 1.28 | 28 | 4.38 | 44 | 12.29 | **4.38** |
| Allergy | 19 | 2.02 | 23 | 3.6 | 27 | 7.54 | **3.6** |
| Venous and lymphatic diseases | 6 | 0.64 | 23 | 3.6 | 25 | 6.98 | **3.6** |
| Chronic pancreas, biliary tract and gallbladder diseases | 15 | 1.6 | 22 | 3.44 | 22 | 6.15 | **3.44** |
| Parkinson and parkinsonism | 17 | 1.81 | 21 | 3.29 | 19 | 5.31 | **3.29** |
| Other respiratory diseases | 16 | 1.7 | 18 | 2.82 | 13 | 3.63 | **2.82** |
| Migraine and facial pain syndromes | 13 | 1.38 | 14 | 2.19 | 19 | 5.31 | **2.19** |
| Other digestive diseases | 3 | 0.32 | 13 | 2.03 | 20 | 5.59 | **2.03** |
| Chronic infectious diseases | 3 | 0.32 | 12 | 1.88 | 8 | 2.23 | 1.88 |
| Chronic ulcer of the skin | 6 | 0.64 | 11 | 1.72 | 20 | 5.59 | 1.72 |
| Inflammatory bowel diseases | 9 | 0.96 | 11 | 1.72 | 10 | 2.79 | 1.72 |
| Epilepsy | 14 | 1.49 | 5 | 0.78 | 5 | 1.4 | 1.4 |
| Blood and blood forming organ diseases | 6 | 0.64 | 8 | 1.25 | 11 | 3.07 | 1.25 |
| Hematological neoplasms | 6 | 0.64 | 6 | 0.94 | 7 | 1.96 | 0.94 |
| Other skin diseases | 3 | 0.32 | 5 | 0.78 | 7 | 1.96 | 0.78 |
| Chronic liver diseases | 2 | 0.21 | 3 | 0.47 | 2 | 0.56 | 0.47 |
| Schizophrenia and delusional diseases | 5 | 0.53 | 2 | 0.31 | 1 | 0.28 | 0.31 |
| Chromosomal abnormalities | 0 | 0 | 1 | 0.16 | 1 | 0.28 | 0.16 |
| Multiple sclerosis | 2 | 0.21 | 1 | 0.16 | 0 | 0 | 0.16 |

*Octogenarians and beyond*

|  | N_  Baseline | Prev_  Baseline | N_  3 years | Prev_  3 years | N_  6 years | Prev_  6 years | N_  9 years | Prev_  9 years | N_  12 years | Prev_  12 years | median |
| --- | --- | --- | --- | --- | --- | --- | --- | --- | --- | --- | --- |
| Hypertension | 770 | 68.75 | 545 | 85.16 | 339 | 90.64 | 198 | 94.29 | 90 | 95.74 | **90.64** |
| Chronic kidney diseases | 652 | 58.21 | 426 | 66.56 | 264 | 70.59 | 156 | 74.29 | 76 | 80.85 | **70.59** |
| Dyslipidemia | 403 | 35.98 | 307 | 47.97 | 202 | 54.01 | 125 | 59.52 | 62 | 65.96 | **54.01** |
| Deafness, hearing impairment | 287 | 25.62 | 260 | 40.62 | 185 | 49.47 | 130 | 61.9 | 80 | 85.11 | **49.47** |
| Colitis and related diseases | 238 | 21.25 | 231 | 36.09 | 178 | 47.59 | 117 | 55.71 | 61 | 64.89 | **47.59** |
| Anemia | 273 | 24.38 | 210 | 32.81 | 155 | 41.44 | 97 | 46.19 | 47 | 50 | **41.44** |
| Cataract and other lens diseases | 107 | 9.55 | 163 | 25.47 | 143 | 38.24 | 111 | 52.86 | 67 | 71.28 | **38.24** |
| Heart failure | 253 | 22.59 | 205 | 32.03 | 136 | 36.36 | 82 | 39.05 | 38 | 40.43 | **36.36** |
| Osteoarthritis and other degenerative joint diseases | 154 | 13.75 | 157 | 24.53 | 129 | 34.49 | 102 | 48.57 | 55 | 58.51 | **34.49** |
| Dementia | 277 | 24.73 | 186 | 29.06 | 129 | 34.49 | 71 | 33.81 | 37 | 39.36 | **33.81** |
| Ischemic heart disease | 281 | 25.09 | 186 | 29.06 | 125 | 33.42 | 71 | 33.81 | 30 | 31.91 | **31.91** |
| Other eye diseases | 97 | 8.66 | 136 | 21.25 | 115 | 30.75 | 76 | 36.19 | 48 | 51.06 | **30.75** |
| Solid neoplasms | 108 | 9.64 | 106 | 16.56 | 93 | 24.87 | 62 | 29.52 | 35 | 37.23 | **24.87** |
| Other musculoskeletal and joint diseases | 122 | 10.89 | 113 | 17.66 | 91 | 24.33 | 74 | 35.24 | 40 | 42.55 | **24.33** |
| Blindness, visual impairment | 118 | 10.54 | 120 | 18.75 | 83 | 22.19 | 64 | 30.48 | 38 | 40.43 | **22.19** |
| Cerebrovascular disease | 150 | 13.39 | 126 | 19.69 | 77 | 20.59 | 46 | 21.9 | 26 | 27.66 | **20.59** |
| Atrial fibrillation | 187 | 16.7 | 131 | 20.47 | 98 | 26.2 | 49 | 23.33 | 19 | 20.21 | **20.47** |
| Osteoporosis | 123 | 10.98 | 103 | 16.09 | 75 | 20.05 | 50 | 23.81 | 32 | 34.04 | **20.05** |
| Glaucoma | 129 | 11.52 | 108 | 16.88 | 71 | 18.98 | 45 | 21.43 | 23 | 24.47 | **18.98** |
| Depression and mood diseases | 117 | 10.45 | 110 | 17.19 | 70 | 18.72 | 48 | 22.86 | 30 | 31.91 | **18.72** |
| Dorsopathies | 85 | 7.59 | 74 | 11.56 | 67 | 17.91 | 52 | 24.76 | 30 | 31.91 | **17.91** |
| Thyroid diseases | 155 | 13.84 | 97 | 15.16 | 56 | 14.97 | 40 | 19.05 | 18 | 19.15 | **15.16** |
| Diabetes | 102 | 9.11 | 72 | 11.25 | 53 | 14.17 | 30 | 14.29 | 17 | 18.09 | **14.17** |
| Esophagus, stomach and duodenum diseases | 56 | 5 | 53 | 8.28 | 47 | 12.57 | 37 | 17.62 | 21 | 22.34 | **12.57** |
| Inflammatory arthropathies | 55 | 4.91 | 49 | 7.66 | 47 | 12.57 | 25 | 11.9 | 20 | 21.28 | **11.9** |
| Obesity | 70 | 6.25 | 66 | 10.31 | 44 | 11.76 | 32 | 15.24 | 16 | 17.02 | **11.76** |
| Other genitourinary diseases | 37 | 3.3 | 39 | 6.09 | 40 | 10.7 | 31 | 14.76 | 27 | 28.72 | **10.7** |
| Prostate diseases | 45 | 4.02 | 48 | 7.5 | 40 | 10.7 | 29 | 13.81 | 11 | 11.7 | **10.7** |
| Autoimmune diseases | 64 | 5.71 | 54 | 8.44 | 39 | 10.43 | 25 | 11.9 | 19 | 20.21 | **10.43** |
| Neurotic, stress-related and somatoform diseases | 35 | 3.12 | 44 | 6.88 | 39 | 10.43 | 39 | 18.57 | 26 | 27.66 | **10.43** |
| Other cardiovascular diseases | 60 | 5.36 | 50 | 7.81 | 35 | 9.36 | 25 | 11.9 | 18 | 19.15 | **9.36** |
| COPD, emphysema, chronic bronchitis | 68 | 6.07 | 52 | 8.12 | 36 | 9.63 | 21 | 10 | 8 | 8.51 | **8.51** |
| Cardiac valve diseases | 39 | 3.48 | 39 | 6.09 | 30 | 8.02 | 20 | 9.52 | 14 | 14.89 | **8.02** |
| Other psychiatric and behavioral diseases | 36 | 3.21 | 32 | 5 | 34 | 9.09 | 18 | 8.57 | 7 | 7.45 | **7.45** |
| Asthma | 56 | 5 | 39 | 6.09 | 24 | 6.42 | 13 | 6.19 | 7 | 7.45 | **6.19** |
| Other neurological diseases | 20 | 1.79 | 20 | 3.12 | 22 | 5.88 | 18 | 8.57 | 17 | 18.09 | **5.88** |
| Chronic ulcer of the skin | 19 | 1.7 | 20 | 3.12 | 20 | 5.35 | 19 | 9.05 | 12 | 12.77 | **5.35** |
| Parkinson and parkinsonism | 18 | 1.61 | 20 | 3.12 | 20 | 5.35 | 15 | 7.14 | 10 | 10.64 | **5.35** |
| Peripheral neuropathy | 20 | 1.79 | 23 | 3.59 | 19 | 5.08 | 20 | 9.52 | 13 | 13.83 | **5.08** |
| Sleep disorders | 19 | 1.7 | 21 | 3.28 | 17 | 4.55 | 12 | 5.71 | 5 | 5.32 | **4.55** |
| Other metabolic diseases | 21 | 1.88 | 21 | 3.28 | 16 | 4.28 | 10 | 4.76 | 18 | 19.15 | **4.28** |
| Bradycardias and conduction diseases | 36 | 3.21 | 16 | 2.5 | 14 | 3.74 | 10 | 4.76 | 5 | 5.32 | **3.74** |
| Peripheral vascular disease | 26 | 2.32 | 19 | 2.97 | 14 | 3.74 | 13 | 6.19 | 11 | 11.7 | **3.74** |
| Venous and lymphatic diseases | 9 | 0.8 | 16 | 2.5 | 14 | 3.74 | 14 | 6.67 | 8 | 8.51 | **3.74** |
| Migraine and facial pain syndromes | 31 | 2.77 | 16 | 2.5 | 13 | 3.48 | 9 | 4.29 | 6 | 6.38 | **3.48** |
| Ear, nose, throat diseases | 7 | 0.62 | 12 | 1.88 | 9 | 2.41 | 12 | 5.71 | 10 | 10.64 | **2.41** |
| Other respiratory diseases | 12 | 1.07 | 11 | 1.72 | 10 | 2.67 | 6 | 2.86 | 2 | 2.13 | **2.13** |
| Hematological neoplasms | 12 | 1.07 | 12 | 1.88 | 7 | 1.87 | 5 | 2.38 | 2 | 2.13 | 1.88 |
| Allergy | 9 | 0.8 | 7 | 1.09 | 7 | 1.87 | 8 | 3.81 | 2 | 2.13 | 1.87 |
| Chronic pancreas, biliary tract and gallbladder diseases | 15 | 1.34 | 8 | 1.25 | 6 | 1.6 | 3 | 1.43 | 2 | 2.13 | 1.43 |
| Inflammatory bowel diseases | 7 | 0.62 | 4 | 0.62 | 5 | 1.34 | 4 | 1.9 | 3 | 3.19 | 1.34 |
| Other digestive diseases | 5 | 0.45 | 8 | 1.25 | 3 | 0.8 | 4 | 1.9 | 6 | 6.38 | 1.25 |
| Blood and blood forming organ diseases | 4 | 0.36 | 5 | 0.78 | 4 | 1.07 | 6 | 2.86 | 4 | 4.26 | 1.07 |
| Chronic infectious diseases | 3 | 0.27 | 4 | 0.62 | 4 | 1.07 | 3 | 1.43 | 3 | 3.19 | 1.07 |
| Epilepsy | 10 | 0.89 | 8 | 1.25 | 6 | 1.6 | 2 | 0.95 | 1 | 1.06 | 1.06 |
| Schizophrenia and delusional diseases | 10 | 0.89 | 6 | 0.94 | 5 | 1.34 | 2 | 0.95 | 0 | 0 | 0.94 |
| Other skin diseases | 0 | 0 | 3 | 0.47 | 1 | 0.27 | 3 | 1.43 | 1 | 1.06 | 0.47 |
| Chronic liver diseases | 1 | 0.09 | 3 | 0.47 | 2 | 0.53 | 0 | 0 | 0 | 0 | 0.09 |
| Chromosomal abnormalities | 0 | 0 | 0 | 0 | 0 | 0 | 0 | 0 | 0 | 0 | 0 |
| Multiple sclerosis | 0 | 0 | 0 | 0 | 0 | 0 | 0 | 0 | 0 | 0 | 0 |
