# Supplementary material for: 12-year evolution of multimorbidity patterns among older adults based on Hidden Markov Models
Source: Aging (Albany NY). 2022 Nov 23;14(24):9805–17. doi: 10.18632/aging.204395 (PMC9831736; doi:10.18632/aging.204395)
Supplement: Supplementary Table 2 [file aging-14-204395-s002.docx]

**Supplementary Table 2. Description of multimorbidity patterns in terms of the top 10 diseases characterizing them by age group and follow-up wave.**

*Sexagenarians*

| **Baseline** | **Unspecific** | | |  |  | **Cardiovascular and anemia** | | |  |  | **Cardio-metabolic** | | |  |  | **Psychiatric-endocrine and sensorial** | | |
| --- | --- | --- | --- | --- | --- | --- | --- | --- | --- | --- | --- | --- | --- | --- | --- | --- | --- | --- |
| Problem | Prev | OE | Exc |  | Problem | Prev | OE | Exc |  | Problem | Prev | OE | Exc |  | Problem | Prev | OE | Exc |
| Dyslipidemia | 52.42 | 1.01 | 84.81 |  | Peripheral vascular disease | 40.00 | 43.47 | 16.67 |  | Cardiac valve diseases | 10.23 | 14.82 | 100.00 |  | Neurotic, stress-related and somatoform diseases | 22.81 | 7.44 | 65.00 |
| Hypertension | 60.16 | 0.98 | 82.81 |  | Heart failure | 40.00 | 30.68 | 11.76 |  | Other cardiovascular diseases | 23.86 | 13.53 | 91.30 |  | Blindness, visual impairment | 5.26 | 6.86 | 60.00 |
| Autoimmune diseases | 2.55 | 0.98 | 82.35 |  | Other metabolic diseases | 40.00 | 28.98 | 11.11 |  | Peripheral vascular disease | 11.36 | 12.35 | 83.33 |  | Glaucoma | 8.77 | 6.35 | 55.56 |
| Venous and lymphatic diseases | 0.82 | 0.97 | 81.82 |  | Other cardiovascular diseases | 40.00 | 22.68 | 8.70 |  | Heart failure | 15.91 | 12.20 | 82.35 |  | Other metabolic diseases | 7.89 | 5.72 | 50.00 |
| Deafness, hearing impairment | 1.64 | 0.89 | 75.00 |  | COPD, emphysema, chronic bronchitis | 60.00 | 19.08 | 7.32 |  | Atrial fibrillation | 18.18 | 6.59 | 44.44 |  | Other neurological diseases | 7.89 | 5.72 | 50.00 |
| Obesity | 13.58 | 0.88 | 73.76 |  | Inflammatory arthropathies | 40.00 | 12.72 | 4.88 |  | Ischemic heart disease | 36.36 | 6.50 | 43.84 |  | Peripheral neuropathy | 4.39 | 5.20 | 45.45 |
| Ear, nose, throat diseases | 1.00 | 0.87 | 73.33 |  | Anemia | 40.00 | 11.59 | 4.44 |  | Diabetes | 37.50 | 5.37 | 36.26 |  | Depression and mood diseases | 41.23 | 4.76 | 41.59 |
| Solid neoplasms | 5.01 | 0.86 | 72.37 |  | Colitis and related diseases | 60.00 | 10.29 | 3.95 |  | COPD, emphysema, chronic bronchitis | 13.64 | 4.34 | 29.27 |  | Other psychiatric and behavioral diseases | 7.02 | 4.58 | 40.00 |
| Other genitourinary diseases | 1.09 | 0.84 | 70.59 |  | Osteoporosis | 20.00 | 8.69 | 3.33 |  | Sleep disorders | 10.23 | 4.17 | 28.13 |  | Thyroid diseases | 30.70 | 3.89 | 33.98 |
| Osteoarthritis and other degenerative joint diseases | 8.02 | 0.81 | 68.22 |  | Chronic kidney diseases | 80.00 | 8.02 | 3.08 |  | Inflammatory arthropathies | 12.50 | 3.98 | 26.83 |  | Osteoporosis | 8.77 | 3.81 | 33.33 |
|  |  |  |  |  |  |  |  |  |  |  |  |  |  |  |  |  |  |  |
| **6 years** | **Unspecific** | | |  |  | **Cardiovascular and anemia** | | |  |  | **Cardio-metabolic** | | |  |  | **Psychiatric-endocrine and sensorial** | | |
| Problem | Prev | OE | Exc |  | Problem | Prev | OE | Exc |  | Problem | Prev | OE | Exc |  | Problem | Prev | OE | Exc |
| Dyslipidemia | 65.15 | 1.01 | 53.05 |  | Peripheral vascular disease | 34.62 | 13.91 | 69.23 |  | Other cardiovascular diseases | 22.03 | 4.80 | 54.17 |  | Glaucoma | 8.87 | 2.44 | 76.32 |
| Hypertension | 67.52 | 0.92 | 48.37 |  | Heart failure | 30.77 | 9.46 | 47.06 |  | Heart failure | 14.41 | 4.43 | 50.00 |  | Blindness, visual impairment | 5.81 | 2.34 | 73.08 |
| Obesity | 14.78 | 0.74 | 38.76 |  | Other cardiovascular diseases | 42.31 | 9.21 | 45.83 |  | Ischemic heart disease | 38.14 | 4.33 | 48.91 |  | Other neurological diseases | 6.42 | 2.31 | 72.41 |
| Solid neoplasms | 10.58 | 0.70 | 36.94 |  | Cardiac valve diseases | 19.23 | 8.04 | 40.00 |  | Cardiac valve diseases | 10.17 | 4.25 | 48.00 |  | Ear, nose, throat diseases | 8.56 | 2.18 | 68.29 |
| Venous and lymphatic diseases | 2.01 | 0.70 | 36.67 |  | Other psychiatric and behavioral diseases | 23.08 | 6.70 | 33.33 |  | Diabetes | 43.22 | 3.76 | 42.50 |  | Depression and mood diseases | 24.46 | 2.10 | 65.57 |
| Chronic pancreas, biliary tract and gallbladder diseases | 2.74 | 0.68 | 35.71 |  | Anemia | 40.38 | 5.48 | 27.27 |  | Atrial fibrillation | 15.25 | 2.80 | 31.58 |  | Osteoporosis | 12.84 | 2.03 | 63.64 |
| Chronic kidney diseases | 8.03 | 0.64 | 33.59 |  | Atrial fibrillation | 28.85 | 5.29 | 26.32 |  | Prostate diseases | 18.64 | 2.71 | 30.56 |  | Thyroid diseases | 22.02 | 2.00 | 62.61 |
| Osteoarthritis and other degenerative joint diseases | 17.52 | 0.62 | 32.32 |  | Other metabolic diseases | 15.38 | 4.73 | 23.53 |  | Sleep disorders | 11.86 | 2.25 | 25.45 |  | Other metabolic diseases | 6.12 | 1.88 | 58.82 |
| Prostate diseases | 4.20 | 0.61 | 31.94 |  | Peripheral neuropathy | 13.46 | 4.26 | 21.21 |  | Peripheral vascular disease | 5.08 | 2.04 | 23.08 |  | Allergy | 11.01 | 1.86 | 58.06 |
| Esophagus, stomach and duodenum diseases | 4.93 | 0.61 | 31.76 |  | Blindness, visual impairment | 9.62 | 3.86 | 19.23 |  | COPD, emphysema, chronic bronchitis | 12.71 | 1.98 | 22.39 |  | Neurotic, stress-related and somatoform diseases | 16.51 | 1.84 | 57.45 |
|  |  |  |  |  |  |  |  |  |  |  |  |  |  |  |  |  |  |  |
| **12 years** | **Unspecific** | | |  |  | **Cardiovascular and anemia** | | |  |  | **Cardio-metabolic** | | |  |  | **Psychiatric-endocrine and sensorial** | | |
| Problem | Prev | OE | Exc |  | Problem | Prev | OE | Exc |  | Problem | Prev | OE | Exc |  | Problem | Prev | OE | Exc |
| Chronic pancreas, biliary tract and gallbladder diseases | 5.24 | 0.98 | 22.22 |  | Peripheral vascular disease | 18.18 | 5.13 | 86.67 |  | Ischemic heart disease | 42.45 | 3.33 | 41.67 |  | Depression and mood diseases | 19.95 | 1.34 | 64.29 |
| Dyslipidemia | 68.06 | 0.96 | 21.67 |  | Other cardiovascular diseases | 29.37 | 4.01 | 67.74 |  | Heart failure | 21.70 | 3.01 | 37.70 |  | Neurotic, stress-related and somatoform diseases | 18.23 | 1.26 | 60.66 |
| Hypertension | 75.92 | 0.96 | 21.58 |  | Heart failure | 25.17 | 3.49 | 59.02 |  | Cardiac valve diseases | 20.75 | 2.98 | 37.29 |  | Thyroid diseases | 16.26 | 1.26 | 60.55 |
| Venous and lymphatic diseases | 6.28 | 0.86 | 19.35 |  | Cardiac valve diseases | 20.28 | 2.91 | 49.15 |  | Diabetes | 38.68 | 2.92 | 36.61 |  | Osteoarthritis and other degenerative joint diseases | 62.07 | 1.25 | 60.14 |
| Solid neoplasms | 19.90 | 0.75 | 16.89 |  | Glaucoma | 18.88 | 2.85 | 48.21 |  | Prostate diseases | 35.85 | 2.71 | 33.93 |  | Deafness, hearing impairment | 20.44 | 1.21 | 58.04 |
| Obesity | 12.04 | 0.54 | 12.17 |  | Other psychiatric and behavioral diseases | 16.78 | 2.84 | 48.00 |  | Other cardiovascular diseases | 16.98 | 2.32 | 29.03 |  | Migraine and facial pain syndromes | 8.37 | 1.20 | 57.63 |
| Other genitourinary diseases | 14.14 | 0.52 | 11.84 |  | Blindness, visual impairment | 13.29 | 2.74 | 46.34 |  | Atrial fibrillation | 22.64 | 2.02 | 25.26 |  | Other musculoskeletal and joint diseases | 36.21 | 1.18 | 56.54 |
| Allergy | 5.24 | 0.49 | 10.99 |  | Atrial fibrillation | 30.07 | 2.68 | 45.26 |  | Obesity | 37.74 | 1.69 | 21.16 |  | Other genitourinary diseases | 31.03 | 1.15 | 55.26 |
| Diabetes | 6.28 | 0.47 | 10.71 |  | COPD, emphysema, chronic bronchitis | 23.08 | 2.44 | 41.25 |  | Sleep disorders | 13.21 | 1.57 | 19.72 |  | Cataract and other lens diseases | 45.81 | 1.13 | 54.39 |
| Esophagus, stomach and duodenum diseases | 7.33 | 0.47 | 10.61 |  | Anemia | 30.77 | 2.43 | 41.12 |  | COPD, emphysema, chronic bronchitis | 13.21 | 1.40 | 17.50 |  | Osteoporosis | 12.56 | 1.10 | 52.58 |

*Septuagenarians*

| **Baseline** | **Unspecific** | | |  |  | **Cardiovascular and diabetes** | | |  |  | **Neuro-vascular and skin-sensorial** | | |  |  | **Neuro-psychiatric and sensorial** | | |
| --- | --- | --- | --- | --- | --- | --- | --- | --- | --- | --- | --- | --- | --- | --- | --- | --- | --- | --- |
| Problem | Prev | OE | Exc |  | Problem | Prev | OE | Exc |  | Problem | Prev | OE | Exc |  | Problem | Prev | OE | Exc |
| Dyslipidemia | 54.59 | 1.07 | 74.84 |  | Other respiratory diseases | 13.16 | 7.72 | 62.50 |  | Venous and lymphatic diseases | 25.00 | 39.13 | 16.67 |  | Parkinson and parkinsonism | 7.32 | 4.04 | 88.24 |
| Sleep disorders | 2.14 | 1.06 | 73.68 |  | Peripheral vascular disease | 13.16 | 7.72 | 62.50 |  | Chronic ulcer of the skin | 25.00 | 39.13 | 16.67 |  | Allergy | 7.80 | 3.86 | 84.21 |
| Hypertension | 74.92 | 0.99 | 69.01 |  | Bradycardias and conduction diseases | 17.11 | 7.65 | 61.90 |  | Other neurological diseases | 50.00 | 17.39 | 7.41 |  | Other psychiatric and behavioral diseases | 6.34 | 3.31 | 72.22 |
| Thyroid diseases | 9.79 | 0.98 | 68.09 |  | Other cardiovascular diseases | 26.32 | 7.49 | 60.61 |  | Blindness, visual impairment | 25.00 | 14.67 | 6.25 |  | Peripheral neuropathy | 6.34 | 3.31 | 72.22 |
| Other digestive diseases | 0.31 | 0.96 | 66.67 |  | Venous and lymphatic diseases | 3.95 | 6.18 | 50.00 |  | Peripheral vascular disease | 25.00 | 14.67 | 6.25 |  | Neurotic, stress-related and somatoform diseases | 9.76 | 3.05 | 66.67 |
| Deafness, hearing impairment | 7.34 | 0.92 | 64.00 |  | Heart failure | 51.32 | 5.81 | 46.99 |  | Parkinson and parkinsonism | 25.00 | 13.81 | 5.88 |  | Depression and mood diseases | 24.39 | 2.86 | 62.50 |
| Glaucoma | 3.98 | 0.89 | 61.90 |  | Cardiac valve diseases | 21.05 | 5.65 | 45.71 |  | Allergy | 25.00 | 12.36 | 5.26 |  | Blindness, visual impairment | 4.88 | 2.86 | 62.50 |
| Chronic kidney diseases | 31.35 | 0.88 | 61.19 |  | Diabetes | 48.68 | 4.44 | 35.92 |  | Dementia | 50.00 | 12.04 | 5.13 |  | Migraine and facial pain syndromes | 3.90 | 2.82 | 61.54 |
| Solid neoplasms | 10.70 | 0.87 | 60.87 |  | Chronic ulcer of the skin | 2.63 | 4.12 | 33.33 |  | Other cardiovascular diseases | 25.00 | 7.11 | 3.03 |  | Dementia | 11.71 | 2.82 | 61.54 |
| Chronic pancreas, biliary tract and gallbladder diseases | 1.38 | 0.86 | 60.00 |  | Other digestive diseases | 1.32 | 4.12 | 33.33 |  | Asthma | 50.00 | 7.01 | 2.99 |  | Other neurological diseases | 7.80 | 2.71 | 59.26 |
|  |  |  |  |  |  |  |  |  |  |  |  |  |  |  |  |  |  |  |
| **6 years** | **Unspecific** | | |  |  | **Cardiovascular and diabetes** | | |  |  | **Neuro-vascular and skin-sensorial** | | |  |  | **Neuro-psychiatric and sensorial** | | |
| Problem | Prev | OE | Exc |  | Problem | Prev | OE | Exc |  | Problem | Prev | OE | Exc |  | Problem | Prev | OE | Exc |
| Dyslipidemia | 68.39 | 1.09 | 26.43 |  | Bradycardias and conduction diseases | 35.38 | 6.46 | 65.71 |  | Chronic ulcer of the skin | 15.09 | 8.77 | 72.73 |  | Dementia | 19.95 | 1.36 | 77.66 |
| Hypertension | 85.16 | 0.98 | 23.70 |  | Other respiratory diseases | 15.38 | 5.46 | 55.56 |  | Parkinson and parkinsonism | 20.75 | 6.32 | 52.38 |  | Deafness, hearing impairment | 24.59 | 1.30 | 74.38 |
| Thyroid diseases | 12.26 | 0.75 | 18.27 |  | Peripheral vascular disease | 20.00 | 4.12 | 41.94 |  | Peripheral neuropathy | 26.42 | 4.69 | 38.89 |  | Ear, nose, throat diseases | 6.28 | 1.30 | 74.19 |
| Chronic pancreas, biliary tract and gallbladder diseases | 2.58 | 0.75 | 18.18 |  | Other cardiovascular diseases | 32.31 | 4.05 | 41.18 |  | Allergy | 15.09 | 4.19 | 34.78 |  | Other psychiatric and behavioral diseases | 10.38 | 1.28 | 73.08 |
| Solid neoplasms | 18.71 | 0.72 | 17.58 |  | Heart failure | 64.62 | 3.79 | 38.53 |  | Peripheral vascular disease | 18.87 | 3.89 | 32.26 |  | Migraine and facial pain syndromes | 2.73 | 1.25 | 71.43 |
| Obesity | 12.90 | 0.72 | 17.39 |  | Cardiac valve diseases | 27.69 | 3.40 | 34.62 |  | Other metabolic diseases | 16.98 | 3.88 | 32.14 |  | Glaucoma | 13.66 | 1.25 | 71.43 |
| Chronic kidney diseases | 29.03 | 0.71 | 17.11 |  | Other digestive diseases | 6.15 | 3.02 | 30.77 |  | Other cardiovascular diseases | 30.19 | 3.78 | 31.37 |  | Other genitourinary diseases | 15.30 | 1.24 | 70.89 |
| Prostate diseases | 7.74 | 0.68 | 16.44 |  | Atrial fibrillation | 55.38 | 2.97 | 30.25 |  | Venous and lymphatic diseases | 13.21 | 3.67 | 30.43 |  | Other eye diseases | 26.50 | 1.22 | 69.78 |
| Osteoarthritis and other degenerative joint diseases | 25.81 | 0.66 | 15.94 |  | Diabetes | 40.00 | 2.87 | 29.21 |  | Other neurological diseases | 18.87 | 3.55 | 29.41 |  | Neurotic, stress-related and somatoform diseases | 12.02 | 1.20 | 68.75 |
| Other genitourinary diseases | 7.74 | 0.63 | 15.19 |  | Ischemic heart disease | 64.62 | 2.79 | 28.38 |  | Blindness, visual impairment | 20.75 | 2.95 | 24.44 |  | Other musculoskeletal and joint diseases | 23.77 | 1.19 | 67.97 |
|  |  |  |  |  |  |  |  |  |  |  |  |  |  |  |  |  |  |  |
| **12 years** | **Unspecific** | | |  |  | **Cardiovascular and diabetes** | | |  |  | **Neuro-vascular and skin-sensorial** | | |  |  | **Neuro-psychiatric and sensorial** | | |
| Problem | Prev | OE | Exc |  | Problem | Prev | OE | Exc |  | Problem | Prev | OE | Exc |  | Problem | Prev | OE | Exc |
| Thyroid diseases | 23.81 | 1.15 | 6.76 |  | Bradycardias and conduction diseases | 33.33 | 4.77 | 36.00 |  | Chronic ulcer of the skin | 13.74 | 2.46 | 90.00 |  | Thyroid diseases | 21.79 | 1.05 | 52.70 |
| Hypertension | 95.24 | 1.03 | 6.04 |  | Venous and lymphatic diseases | 29.63 | 4.24 | 32.00 |  | Parkinson and parkinsonism | 12.21 | 2.30 | 84.21 |  | Sleep disorders | 5.59 | 1.05 | 52.63 |
| Dyslipidemia | 66.67 | 0.93 | 5.47 |  | Other respiratory diseases | 14.81 | 4.08 | 30.77 |  | Peripheral vascular disease | 12.21 | 2.08 | 76.19 |  | Dyslipidemia | 73.18 | 1.02 | 51.17 |
| Osteoarthritis and other degenerative joint diseases | 42.86 | 0.76 | 4.48 |  | Peripheral vascular disease | 18.52 | 3.16 | 23.81 |  | Other cardiovascular diseases | 29.77 | 1.94 | 70.91 |  | Cataract and other lens diseases | 62.57 | 1.01 | 50.45 |
| Asthma | 9.52 | 0.73 | 4.26 |  | Diabetes | 48.15 | 2.61 | 19.70 |  | Other psychiatric and behavioral diseases | 21.37 | 1.91 | 70.00 |  | Esophagus, stomach and duodenum diseases | 17.88 | 1.00 | 50.00 |
| COPD, emphysema, chronic bronchitis | 9.52 | 0.68 | 4.00 |  | Other cardiovascular diseases | 37.04 | 2.41 | 18.18 |  | Chronic pancreas, biliary tract and gallbladder diseases | 11.45 | 1.86 | 68.18 |  | Deafness, hearing impairment | 45.25 | 0.98 | 49.09 |
| Solid neoplasms | 28.57 | 0.66 | 3.85 |  | Cardiac valve diseases | 37.04 | 2.29 | 17.24 |  | Allergy | 13.74 | 1.82 | 66.67 |  | Chronic kidney diseases | 50.28 | 0.97 | 48.39 |
| Dorsopathies | 14.29 | 0.58 | 3.41 |  | COPD, emphysema, chronic bronchitis | 29.63 | 2.12 | 16.00 |  | Other neurological diseases | 19.08 | 1.80 | 65.79 |  | Osteoarthritis and other degenerative joint diseases | 53.63 | 0.96 | 47.76 |
| Obesity | 9.52 | 0.48 | 2.82 |  | Migraine and facial pain syndromes | 11.11 | 2.09 | 15.79 |  | Other digestive diseases | 9.92 | 1.78 | 65.00 |  | Hypertension | 88.27 | 0.95 | 47.73 |
| Chronic kidney diseases | 23.81 | 0.46 | 2.69 |  | Prostate diseases | 25.93 | 1.97 | 14.89 |  | Other metabolic diseases | 21.37 | 1.74 | 63.64 |  | Glaucoma | 16.76 | 0.95 | 47.62 |

*Octogenarians and beyond*

| **Baseline** | **Unspecific** | | |  |  | **Respiratory-circulatory and skin** | | |  |  | **Cardio-respiratory and Neurological** | | |  |  | **Neuro-sensorial** | | |
| --- | --- | --- | --- | --- | --- | --- | --- | --- | --- | --- | --- | --- | --- | --- | --- | --- | --- | --- |
| Problem | Prev | OE | Exc |  | Problem | Prev | OE | Exc |  | Problem | Prev | OE | Exc |  | Problem | Prev | OE | Exc |
| Hypertension | 72.28 | 1.05 | 76.88 |  | Venous and lymphatic diseases | 42.86 | 53.33 | 66.67 |  | Bradycardias and conduction diseases | 9.80 | 3.05 | 69.44 |  | Other digestive diseases | 9.38 | 21.00 | 60.00 |
| Dyslipidemia | 37.73 | 1.05 | 76.67 |  | Chronic ulcer of the skin | 57.14 | 33.68 | 42.11 |  | Asthma | 14.90 | 2.98 | 67.86 |  | Other neurological diseases | 21.88 | 12.25 | 35.00 |
| Obesity | 6.11 | 0.98 | 71.43 |  | Other respiratory diseases | 28.57 | 26.67 | 33.33 |  | COPD, emphysema, chronic bronchitis | 18.04 | 2.97 | 67.65 |  | Ear, nose, throat diseases | 6.25 | 10.00 | 28.57 |
| Chronic kidney diseases | 56.78 | 0.98 | 71.32 |  | Peripheral vascular disease | 28.57 | 12.31 | 15.38 |  | Other metabolic diseases | 5.49 | 2.93 | 66.67 |  | Parkinson and parkinsonism | 15.63 | 9.72 | 27.78 |
| Thyroid diseases | 13.43 | 0.97 | 70.97 |  | Other metabolic diseases | 21.43 | 11.43 | 14.29 |  | Migraine and facial pain syndromes | 7.45 | 2.69 | 61.29 |  | Peripheral vascular disease | 21.88 | 9.42 | 26.92 |
| Solid neoplasms | 9.28 | 0.96 | 70.37 |  | Inflammatory arthropathies | 35.71 | 7.27 | 9.09 |  | Other respiratory diseases | 2.75 | 2.56 | 58.33 |  | Other cardiovascular diseases | 46.88 | 8.75 | 25.00 |
| Dementia | 23.57 | 0.95 | 69.68 |  | Other cardiovascular diseases | 35.71 | 6.67 | 8.33 |  | Sleep disorders | 4.31 | 2.54 | 57.89 |  | Neurotic, stress-related and somatoform diseases | 25.00 | 8.00 | 22.86 |
| Cataract and other lens diseases | 8.79 | 0.92 | 67.29 |  | COPD, emphysema, chronic bronchitis | 28.57 | 4.71 | 5.88 |  | Chronic ulcer of the skin | 3.92 | 2.31 | 52.63 |  | Migraine and facial pain syndromes | 18.75 | 6.77 | 19.35 |
| Deafness, hearing impairment | 23.57 | 0.92 | 67.25 |  | Parkinson and parkinsonism | 7.14 | 4.44 | 5.56 |  | Neurotic, stress-related and somatoform diseases | 7.06 | 2.26 | 51.43 |  | Sleep disorders | 9.38 | 5.53 | 15.79 |
| Osteoarthritis and other degenerative joint diseases | 12.58 | 0.91 | 66.88 |  | Asthma | 21.43 | 4.29 | 5.36 |  | Parkinson and parkinsonism | 3.53 | 2.20 | 50.00 |  | Peripheral neuropathy | 9.38 | 5.25 | 15.00 |
|  |  |  |  |  |  |  |  |  |  |  |  |  |  |  |  |  |  |  |
| **3 years** | **Unspecific** | | |  |  | **Respiratory-circulatory and skin** | | |  |  | **Cardio-respiratory and Neurological** | | |  |  | **Neuro-sensorial** | | |
| Problem | Prev | OE | Exc |  | Problem | Prev | OE | Exc |  | Problem | Prev | OE | Exc |  | Problem | Prev | OE | Exc |
| Dyslipidemia | 51.34 | 1.07 | 49.84 |  | Venous and lymphatic diseases | 41.67 | 16.67 | 62.50 |  | Other psychiatric and behavioral diseases | 8.21 | 1.64 | 71.88 |  | Other neurological diseases | 26.32 | 8.42 | 50.00 |
| Hypertension | 87.25 | 1.02 | 47.71 |  | Other respiratory diseases | 25.00 | 14.55 | 54.55 |  | Blindness, visual impairment | 30.36 | 1.62 | 70.83 |  | Peripheral vascular disease | 21.05 | 7.09 | 42.11 |
| Dorsopathies | 10.74 | 0.93 | 43.24 |  | Chronic ulcer of the skin | 37.50 | 12.00 | 45.00 |  | COPD, emphysema, chronic bronchitis | 12.86 | 1.58 | 69.23 |  | Sleep disorders | 21.05 | 6.42 | 38.10 |
| Osteoarthritis and other degenerative joint diseases | 22.48 | 0.92 | 42.68 |  | Peripheral vascular disease | 25.00 | 8.42 | 31.58 |  | Depression and mood diseases | 26.43 | 1.54 | 67.27 |  | Other digestive diseases | 7.89 | 6.32 | 37.50 |
| Thyroid diseases | 13.76 | 0.91 | 42.27 |  | Asthma | 41.67 | 6.84 | 25.64 |  | Asthma | 9.29 | 1.52 | 66.67 |  | Parkinson and parkinsonism | 18.42 | 5.89 | 35.00 |
| Chronic kidney diseases | 59.73 | 0.90 | 41.78 |  | COPD, emphysema, chronic bronchitis | 45.83 | 5.64 | 21.15 |  | Other genitourinary diseases | 8.93 | 1.47 | 64.10 |  | Peripheral neuropathy | 21.05 | 5.86 | 34.78 |
| Cataract and other lens diseases | 22.82 | 0.90 | 41.72 |  | Other cardiovascular diseases | 37.50 | 4.80 | 18.00 |  | Cerebrovascular disease | 28.57 | 1.45 | 63.49 |  | Ear, nose, throat diseases | 10.53 | 5.61 | 33.33 |
| Deafness, hearing impairment | 36.24 | 0.89 | 41.54 |  | Other metabolic diseases | 12.50 | 3.81 | 14.29 |  | Bradycardias and conduction diseases | 3.57 | 1.43 | 62.50 |  | Other cardiovascular diseases | 39.47 | 5.05 | 30.00 |
| Obesity | 9.06 | 0.88 | 40.91 |  | Other genitourinary diseases | 20.83 | 3.42 | 12.82 |  | Heart failure | 45.36 | 1.42 | 61.95 |  | Migraine and facial pain syndromes | 10.53 | 4.21 | 25.00 |
| Dementia | 25.50 | 0.88 | 40.86 |  | Other digestive diseases | 4.17 | 3.33 | 12.50 |  | Other eye diseases | 29.64 | 1.39 | 61.03 |  | Other metabolic diseases | 13.16 | 4.01 | 23.81 |
|  |  |  |  |  |  |  |  |  |  |  |  |  |  |  |  |  |  |  |
| **6 years** | **Unspecific** | | |  |  | **Respiratory-circulatory and skin** | | |  |  | **Cardio-respiratory and Neurological** | | |  |  | **Neuro-sensorial** | | |
| Problem | Prev | OE | Exc |  | Problem | Prev | OE | Exc |  | Problem | Prev | OE | Exc |  | Problem | Prev | OE | Exc |
| Dyslipidemia | 60.55 | 1.12 | 32.67 |  | Venous and lymphatic diseases | 41.94 | 11.20 | 92.86 |  | Blindness, visual impairment | 31.28 | 1.41 | 73.49 |  | Parkinson and parkinsonism | 28.21 | 5.27 | 55.00 |
| Hypertension | 91.74 | 1.01 | 29.50 |  | Peripheral vascular disease | 29.03 | 7.76 | 64.29 |  | Glaucoma | 26.15 | 1.38 | 71.83 |  | Bradycardias and conduction diseases | 17.95 | 4.79 | 50.00 |
| Osteoarthritis and other degenerative joint diseases | 34.86 | 1.01 | 29.46 |  | Chronic ulcer of the skin | 35.48 | 6.64 | 55.00 |  | Other psychiatric and behavioral diseases | 11.79 | 1.30 | 67.65 |  | Peripheral neuropathy | 23.08 | 4.54 | 47.37 |
| Deafness, hearing impairment | 48.62 | 0.98 | 28.65 |  | Other respiratory diseases | 16.13 | 6.03 | 50.00 |  | Atrial fibrillation | 32.31 | 1.23 | 64.29 |  | Sleep disorders | 20.51 | 4.51 | 47.06 |
| Chronic kidney diseases | 63.30 | 0.90 | 26.14 |  | Asthma | 32.26 | 5.03 | 41.67 |  | Depression and mood diseases | 23.08 | 1.23 | 64.29 |  | Other neurological diseases | 23.08 | 3.92 | 40.91 |
| Dementia | 30.28 | 0.88 | 25.58 |  | Other cardiovascular diseases | 41.94 | 4.48 | 37.14 |  | Cerebrovascular disease | 25.13 | 1.22 | 63.64 |  | Peripheral vascular disease | 12.82 | 3.42 | 35.71 |
| Dorsopathies | 14.68 | 0.82 | 23.88 |  | Other digestive diseases | 3.23 | 4.02 | 33.33 |  | Other eye diseases | 36.41 | 1.18 | 61.74 |  | Other digestive diseases | 2.56 | 3.20 | 33.33 |
| Obesity | 9.17 | 0.78 | 22.73 |  | Other metabolic diseases | 16.13 | 3.77 | 31.25 |  | Other musculoskeletal and joint diseases | 28.72 | 1.18 | 61.54 |  | Ear, nose, throat diseases | 7.69 | 3.20 | 33.33 |
| Solid neoplasms | 19.27 | 0.77 | 22.58 |  | COPD, emphysema, chronic bronchitis | 35.48 | 3.69 | 30.56 |  | Migraine and facial pain syndromes | 4.10 | 1.18 | 61.54 |  | Other cardiovascular diseases | 28.21 | 3.01 | 31.43 |
| Cataract and other lens diseases | 28.44 | 0.74 | 21.68 |  | Other genitourinary diseases | 38.71 | 3.62 | 30.00 |  | Colitis and related diseases | 55.38 | 1.16 | 60.67 |  | Neurotic, stress-related and somatoform diseases | 28.21 | 2.70 | 28.21 |
|  |  |  |  |  |  |  |  |  |  |  |  |  |  |  |  |  |  |  |
| **9 years** | **Unspecific** | | |  |  | **Respiratory-circulatory and skin** | | |  |  | **Cardio-respiratory and Neurological** | | |  |  | **Neuro-sensorial** | | |
| Problem | Prev | OE | Exc |  | Problem | Prev | OE | Exc |  | Problem | Prev | OE | Exc |  | Problem | Prev | OE | Exc |
| Diabetes | 15.15 | 1.06 | 16.67 |  | Venous and lymphatic diseases | 54.17 | 8.13 | 92.86 |  | Other psychiatric and behavioral diseases | 13.45 | 1.57 | 88.89 |  | Parkinson and parkinsonism | 32.35 | 4.53 | 73.33 |
| Esophagus, stomach and duodenum diseases | 18.18 | 1.03 | 16.22 |  | Peripheral vascular disease | 33.33 | 5.38 | 61.54 |  | Glaucoma | 26.89 | 1.25 | 71.11 |  | Other neurological diseases | 29.41 | 3.43 | 55.56 |
| Dyslipidemia | 60.61 | 1.02 | 16.00 |  | Chronic ulcer of the skin | 41.67 | 4.61 | 52.63 |  | Blindness, visual impairment | 36.13 | 1.19 | 67.19 |  | Bradycardias and conduction diseases | 14.71 | 3.09 | 50.00 |
| Dementia | 33.33 | 0.99 | 15.49 |  | Other digestive diseases | 8.33 | 4.38 | 50.00 |  | Other eye diseases | 42.86 | 1.18 | 67.11 |  | Peripheral neuropathy | 29.41 | 3.09 | 50.00 |
| Hypertension | 90.91 | 0.96 | 15.15 |  | Other respiratory diseases | 12.50 | 4.38 | 50.00 |  | Migraine and facial pain syndromes | 5.04 | 1.18 | 66.67 |  | Inflammatory arthropathies | 32.35 | 2.72 | 44.00 |
| Chronic kidney diseases | 69.70 | 0.94 | 14.74 |  | Other cardiovascular diseases | 45.83 | 3.85 | 44.00 |  | Obesity | 17.65 | 1.16 | 65.63 |  | Other cardiovascular diseases | 32.35 | 2.72 | 44.00 |
| Anemia | 42.42 | 0.92 | 14.43 |  | Asthma | 20.83 | 3.37 | 38.46 |  | Cardiac valve diseases | 10.92 | 1.15 | 65.00 |  | Sleep disorders | 14.71 | 2.57 | 41.67 |
| Deafness, hearing impairment | 54.55 | 0.88 | 13.85 |  | COPD, emphysema, chronic bronchitis | 29.17 | 2.92 | 33.33 |  | Depression and mood diseases | 26.05 | 1.14 | 64.58 |  | Peripheral vascular disease | 14.71 | 2.38 | 38.46 |
| Thyroid diseases | 15.15 | 0.80 | 12.50 |  | Other neurological diseases | 20.83 | 2.43 | 27.78 |  | Solid neoplasms | 33.61 | 1.14 | 64.52 |  | Migraine and facial pain syndromes | 8.82 | 2.06 | 33.33 |
| Osteoarthritis and other degenerative joint diseases | 36.36 | 0.75 | 11.76 |  | Other genitourinary diseases | 33.33 | 2.26 | 25.81 |  | Cataract and other lens diseases | 59.66 | 1.13 | 63.96 |  | Ear, nose, throat diseases | 11.76 | 2.06 | 33.33 |
|  |  |  |  |  |  |  |  |  |  |  |  |  |  |  |  |  |  |  |
| **12 years** | **Unspecific** | | |  |  | **Respiratory-circulatory and skin** | | |  |  | **Cardio-respiratory and Neurological** | | |  |  | **Neuro-sensorial** | | |
| Problem | Prev | OE | Exc |  | Problem | Prev | OE | Exc |  | Problem | Prev | OE | Exc |  | Problem | Prev | OE | Exc |
| Cerebrovascular disease | 33.33 | 1.21 | 7.69 |  | Venous and lymphatic diseases | 53.33 | 6.27 | 100.00 |  | Other psychiatric and behavioral diseases | 12.24 | 1.64 | 85.71 |  | Ear, nose, throat diseases | 25.00 | 2.35 | 60.00 |
| Hypertension | 100.00 | 1.04 | 6.67 |  | Other respiratory diseases | 13.33 | 6.27 | 100.00 |  | Diabetes | 24.49 | 1.35 | 70.59 |  | Parkinson and parkinsonism | 25.00 | 2.35 | 60.00 |
| Deafness, hearing impairment | 83.33 | 0.98 | 6.25 |  | Chronic ulcer of the skin | 60.00 | 4.70 | 75.00 |  | Cardiac valve diseases | 18.37 | 1.23 | 64.29 |  | Bradycardias and conduction diseases | 12.50 | 2.35 | 60.00 |
| Solid neoplasms | 33.33 | 0.90 | 5.71 |  | Peripheral vascular disease | 53.33 | 4.56 | 72.73 |  | Thyroid diseases | 22.45 | 1.17 | 61.11 |  | Peripheral neuropathy | 29.17 | 2.11 | 53.85 |
| Thyroid diseases | 16.67 | 0.87 | 5.56 |  | Other cardiovascular diseases | 60.00 | 3.13 | 50.00 |  | Glaucoma | 28.57 | 1.17 | 60.87 |  | Other neurological diseases | 37.50 | 2.07 | 52.94 |
| Dementia | 33.33 | 0.85 | 5.41 |  | Asthma | 20.00 | 2.69 | 42.86 |  | Cataract and other lens diseases | 81.63 | 1.15 | 59.70 |  | Migraine and facial pain syndromes | 12.50 | 1.96 | 50.00 |
| Autoimmune diseases | 16.67 | 0.82 | 5.26 |  | COPD, emphysema, chronic bronchitis | 20.00 | 2.35 | 37.50 |  | Other eye diseases | 57.14 | 1.12 | 58.33 |  | Other digestive diseases | 12.50 | 1.96 | 50.00 |
| Chronic kidney diseases | 66.67 | 0.82 | 5.26 |  | Heart failure | 86.67 | 2.14 | 34.21 |  | Blindness, visual impairment | 44.90 | 1.11 | 57.89 |  | Neurotic, stress-related and somatoform diseases | 50.00 | 1.81 | 46.15 |
| Other musculoskeletal and joint diseases | 33.33 | 0.78 | 5.00 |  | Atrial fibrillation | 40.00 | 1.98 | 31.58 |  | Atrial fibrillation | 22.45 | 1.11 | 57.89 |  | Prostate diseases | 20.83 | 1.78 | 45.45 |
| Dyslipidemia | 50.00 | 0.76 | 4.84 |  | Parkinson and parkinsonism | 20.00 | 1.88 | 30.00 |  | Obesity | 18.37 | 1.08 | 56.25 |  | Inflammatory arthropathies | 37.50 | 1.76 | 45.00 |
