# Supplementary material for: 12-year evolution of multimorbidity patterns among older adults based on Hidden Markov Models
Source: Aging (Albany NY). 2022 Nov 23;14(24):9805–17. doi: 10.18632/aging.204395 (PMC9831736; doi:10.18632/aging.204395)
Supplement: Supplementary Table 3 [file aging-14-204395-s003.docx]

**Supplementary Table 3. Description of multimorbidity patterns in terms of sociodemographic, clinical and functional characteristics by age group and follow-up wave.**

*Sexagenarians*

| **Baseline** | | | | | | | |  | |  | |
| --- | --- | --- | --- | --- | --- | --- | --- | --- | --- | --- | --- |
|  | **UNSP N=1097** | **CV & ANEMIA N=5** | **CARDIO-META N=88** | **PSY-ENDOC & SENS N=114** | p.overall | N |  | |  | |  |
| Age | 62.9 (2.88) | 65.3 (2.75) | 64.4 (2.79) | 63.7 (2.90) | <0.001 | 1303 |  | |  | |  |
| Sex: |  |  |  |  | 0.003 | 1304 |  | |  | |  |
| Men | 477 (43.5%) | 2 (40.0%) | 52 (59.1%) | 38 (33.3%) |  |  |  | |  | |  |
| Women | 620 (56.5%) | 3 (60.0%) | 36 (40.9%) | 76 (66.7%) |  |  |  | |  | |  |
| Education: |  |  |  |  | 0.065 | 1302 |  | |  | |  |
| Elementary | 75 (6.84%) | 1 (20.0%) | 9 (10.3%) | 8 (7.02%) |  |  |  | |  | |  |
| High school | 465 (42.4%) | 4 (80.0%) | 46 (52.9%) | 46 (40.4%) |  |  |  | |  | |  |
| University | 556 (50.7%) | 0 (0.00%) | 32 (36.8%) | 60 (52.6%) |  |  |  | |  | |  |
| # chronic diseases | 2.23 (1.28) | 9.00 (2.35) | 5.52 (1.89) | 5.00 (1.58) | <0.001 | 1304 |  | |  | |  |
| # drugs | 2.11 (2.18) | 10.0 (4.00) | 6.25 (3.61) | 4.94 (3.25) | <0.001 | 1300 |  | |  | |  |
| Walking speed | 1.29 (0.29) | 0.82 (0.58) | 1.06 (0.40) | 1.15 (0.32) | <0.001 | 1290 |  | |  | |  |
| MMSE | 29.3 (1.43) | 29.0 (1.73) | 28.9 (2.09) | 29.3 (0.98) | 0.100 | 1280 |  | |  | |  |
|  |  |  |  |  |  |  |  | |  | |  |
| **6 years** | | | | | | | | | | | |
|  | **UNSP N=548** | **CV & ANEMIA N=52** | **CARDIO-META N=118** | **PSY-ENDOC & SENS N=327** | **DROPOUT N=181** | **DEATH N=78** | p.overall | | N | |  |
| Age | 62.5 (2.80) | 64.5 (2.78) | 63.8 (2.92) | 63.1 (2.91) | 63.1 (2.93) | 64.3 (2.86) | <0.001 | | 1303 | |  |
| Sex: |  |  |  |  |  |  | <0.001 | | 1304 | |  |
| Men | 244 (44.5%) | 22 (42.3%) | 76 (64.4%) | 100 (30.6%) | 77 (42.5%) | 50 (64.1%) |  | |  | |  |
| Women | 304 (55.5%) | 30 (57.7%) | 42 (35.6%) | 227 (69.4%) | 104 (57.5%) | 28 (35.9%) |  | |  | |  |
| Education: |  |  |  |  |  |  | 0.001 | | 1302 | |  |
| Elementary | 33 (6.02%) | 1 (1.92%) | 7 (5.93%) | 20 (6.12%) | 18 (10.1%) | 14 (17.9%) |  | |  | |  |
| High school | 230 (42.0%) | 32 (61.5%) | 50 (42.4%) | 133 (40.7%) | 78 (43.6%) | 38 (48.7%) |  | |  | |  |
| University | 285 (52.0%) | 19 (36.5%) | 61 (51.7%) | 174 (53.2%) | 83 (46.4%) | 26 (33.3%) |  | |  | |  |
| # chronic diseases | 3.02 (1.35) | 11.3 (2.68) | 6.36 (1.95) | 6.39 (1.94) | . (.) | . (.) | <0.001 | | 1045 | |  |
| # drugs | 2.62 (2.22) | 8.87 (3.89) | 6.18 (3.73) | 5.10 (3.26) | . (.) | . (.) | <0.001 | | 1028 | |  |
| Walking speed | 1.28 (0.30) | 0.93 (0.42) | 1.16 (0.37) | 1.12 (0.38) | . (.) | . (.) | <0.001 | | 1040 | |  |
| MMSE | 28.8 (1.76) | 28.1 (1.84) | 28.7 (1.25) | 28.8 (1.31) | . (.) | . (.) | 0.045 | | 1030 | |  |
|  |  |  |  |  |  |  |  | |  | |  |
| **12 years** | | | | | | | | | | | |
|  | **UNSP N=191** | **CV & ANEMIA N=143** | **CARDIO-META N=106** | **PSY-ENDOC & SENS N=406** | **DROPOUT N=304** | **DEATH N=154** | p.overall | | N | |  |
| Age | 62.3 (2.72) | 63.8 (2.90) | 63.4 (2.96) | 62.7 (2.83) | 63.0 (2.90) | 64.2 (2.88) | <0.001 | | 1303 | |  |
| Sex: |  |  |  |  |  |  | <0.001 | | 1304 | |  |
| Men | 85 (44.5%) | 53 (37.1%) | 75 (70.8%) | 130 (32.0%) | 137 (45.1%) | 89 (57.8%) |  | |  | |  |
| Women | 106 (55.5%) | 90 (62.9%) | 31 (29.2%) | 276 (68.0%) | 167 (54.9%) | 65 (42.2%) |  | |  | |  |
| Education: |  |  |  |  |  |  | 0.005 | | 1302 | |  |
| Elementary | 12 (6.28%) | 10 (6.99%) | 7 (6.60%) | 16 (3.94%) | 31 (10.3%) | 17 (11.0%) |  | |  | |  |
| High school | 80 (41.9%) | 61 (42.7%) | 39 (36.8%) | 166 (40.9%) | 143 (47.4%) | 72 (46.8%) |  | |  | |  |
| University | 99 (51.8%) | 72 (50.3%) | 60 (56.6%) | 224 (55.2%) | 128 (42.4%) | 65 (42.2%) |  | |  | |  |
| # chronic diseases | 3.77 (1.29) | 13.0 (2.83) | 8.20 (2.06) | 7.53 (2.10) | . (.) | . (.) | <0.001 | | 846 | |  |
| # drugs | 2.83 (2.07) | 9.23 (5.04) | 6.12 (3.43) | 4.62 (3.04) | . (.) | . (.) | <0.001 | | 844 | |  |
| Walking speed | 1.24 (0.30) | 0.89 (0.36) | 1.07 (0.33) | 1.08 (0.34) | . (.) | . (.) | <0.001 | | 845 | |  |
| MMSE | 28.7 (1.59) | 28.4 (2.04) | 28.0 (3.03) | 28.5 (2.37) | . (.) | . (.) | 0.139 | | 840 | |  |

Unspecific (USP); Cardiovascular and anemia (CV & ANEMIA); Cardio-metabolic (CARDIO-META) and Psychiatric-endocrine and sensorial (PSY-ENDOC & SENS).

*Septuagenarians*

| **Baseline** | | | | | | |  |  |  |
| --- | --- | --- | --- | --- | --- | --- | --- | --- | --- |
|  | **UNSP N=654** | **CV & DIAB N=76** | **NEUROVASC & SKIN N=4** | **NEUROPSY & SENS N=205** | p.overall | N |  |  |  |
| Age | 75.1 (2.99) | 75.6 (2.98) | 77.0 (3.10) | 75.8 (2.96) | 0.025 | 937 |  |  |  |
| Sex: |  |  |  |  | 0.007 | 939 |  |  |  |
| Men | 231 (35.3%) | 41 (53.9%) | 1 (25.0%) | 68 (33.2%) |  |  |  |  |  |
| Women | 423 (64.7%) | 35 (46.1%) | 3 (75.0%) | 137 (66.8%) |  |  |  |  |  |
| Education: |  |  |  |  | 0.650 | 933 |  |  |  |
| Elementary | 108 (16.6%) | 16 (21.1%) | 1 (25.0%) | 25 (12.4%) |  |  |  |  |  |
| High school | 357 (54.8%) | 42 (55.3%) | 2 (50.0%) | 113 (56.2%) |  |  |  |  |  |
| University | 187 (28.7%) | 18 (23.7%) | 1 (25.0%) | 63 (31.3%) |  |  |  |  |  |
| # chronic diseases | 3.24 (1.44) | 7.42 (2.65) | 10.5 (2.65) | 6.16 (1.73) | <0.001 | 939 |  |  |  |
| # drugs | 3.30 (2.55) | 7.29 (4.02) | 10.0 (2.31) | 6.69 (3.76) | <0.001 | 938 |  |  |  |
| Walking speed | 1.08 (0.34) | 0.78 (0.40) | 0.29 (0.49) | 0.83 (0.40) | <0.001 | 916 |  |  |  |
| MMSE | 28.7 (2.02) | 28.8 (1.30) | 19.7 (17.0) | 27.3 (5.68) | <0.001 | 907 |  |  |  |
|  |  |  |  |  |  |  |  |  |  |
| **6 years** | | | | | | | | | |
|  | **UNSP N=155** | **CV & DIAB N=65** | **NEUROVASC & SKIN N=53** | **NEUROPSY & SENS N=366** | **DROPOUT N=124** | **DEATH N=176** | p.overall | N |  |
| age | 74.3 (2.84) | 75.2 (3.01) | 76.0 (2.96) | 75.2 (2.97) | 76.1 (2.93) | 75.7 (2.99) | <0.001 | 937 |  |
| sex: |  |  |  |  |  |  | 0.001 | 939 |  |
| Men | 53 (34.2%) | 36 (55.4%) | 21 (39.6%) | 110 (30.1%) | 43 (34.7%) | 78 (44.3%) |  |  |  |
| Women | 102 (65.8%) | 29 (44.6%) | 32 (60.4%) | 256 (69.9%) | 81 (65.3%) | 98 (55.7%) |  |  |  |
| education: |  |  |  |  |  |  | 0.084 | 933 |  |
| Elementary | 17 (11.0%) | 15 (23.1%) | 4 (7.55%) | 59 (16.1%) | 22 (18.0%) | 33 (19.2%) |  |  |  |
| High school | 87 (56.1%) | 28 (43.1%) | 31 (58.5%) | 197 (53.8%) | 74 (60.7%) | 97 (56.4%) |  |  |  |
| University | 51 (32.9%) | 22 (33.8%) | 18 (34.0%) | 110 (30.1%) | 26 (21.3%) | 42 (24.4%) |  |  |  |
| # chronic diseases | 3.95 (1.49) | 10.5 (2.98) | 13.2 (2.91) | 8.00 (2.30) | . (.) | . (.) | <0.001 | 639 |  |
| # drugs | 3.01 (2.20) | 8.77 (3.68) | 9.68 (3.79) | 6.41 (3.61) | . (.) | . (.) | <0.001 | 638 |  |
| Walking speed | 1.00 (0.33) | 0.73 (0.43) | 0.55 (0.35) | 0.75 (0.42) | . (.) | . (.) | <0.001 | 632 |  |
| MMSE | 27.7 (2.83) | 27.8 (2.65) | 27.3 (2.11) | 26.3 (5.06) | . (.) | . (.) | 0.002 | 605 |  |
|  |  |  |  |  |  |  |  |  |  |
| **12 years** | | | | | | | | | |
|  | **UNSP N=21** | **CV & DIAB N=27** | **NEUROVASC & SKIN N=131** | **NEUROPSY & SENS N=179** | **DROPOUT N=224** | **DEATH N=357** | p.overall | N |  |
| age | 73.2 (2.27) | 74.4 (2.84) | 75.1 (2.96) | 74.4 (2.85) | 75.5 (3.00) | 75.9 (2.94) | <0.001 | 937 |  |
| sex: |  |  |  |  |  |  | <0.001 | 939 |  |
| Men | 8 (38.1%) | 15 (55.6%) | 33 (25.2%) | 57 (31.8%) | 66 (29.5%) | 162 (45.4%) |  |  |  |
| Women | 13 (61.9%) | 12 (44.4%) | 98 (74.8%) | 122 (68.2%) | 158 (70.5%) | 195 (54.6%) |  |  |  |
| education: |  |  |  |  |  |  | 0.069 | 933 |  |
| Elementary | 2 (9.52%) | 8 (29.6%) | 20 (15.3%) | 18 (10.1%) | 37 (16.7%) | 65 (18.4%) |  |  |  |
| High school | 10 (47.6%) | 13 (48.1%) | 67 (51.1%) | 99 (55.3%) | 126 (56.8%) | 199 (56.4%) |  |  |  |
| University | 9 (42.9%) | 6 (22.2%) | 44 (33.6%) | 62 (34.6%) | 59 (26.6%) | 89 (25.2%) |  |  |  |
| # chronic diseases | 4.67 (1.39) | 12.2 (2.92) | 16.2 (3.88) | 9.88 (2.39) | . (.) | . (.) | <0.001 | 358 |  |
| # drugs | 3.57 (2.23) | 8.30 (4.81) | 9.65 (5.05) | 6.14 (3.28) | . (.) | . (.) | <0.001 | 356 |  |
| Walking speed | 1.01 (0.31) | 0.74 (0.40) | 0.47 (0.37) | 0.75 (0.39) | . (.) | . (.) | <0.001 | 355 |  |
| MMSE | 28.0 (1.45) | 27.0 (2.62) | 24.6 (6.02) | 25.7 (6.05) | . (.) | . (.) | 0.029 | 345 |  |

Unspecific (USP); Cardiovascular and diabetes (CV & DIAB); Neuro-vascular and skin-sensorial (NEUROVASC & SKIN); and Neuro-psychiatric and sensorial (NEUROPSY & SENS).

*Octogenarians and beyond*

| **Baseline** | | | | | | |  |  |  |
| --- | --- | --- | --- | --- | --- | --- | --- | --- | --- |
|  | **UNSP N=819** | **RESP-CIRCULA & SKIN N=14** | **CARDIORESP & NEURO N=255** | **NEURO-SENS N=32** | p.overall | N |  |  |  |
| Age | 87.8 (5.17) | 85.8 (4.57) | 88.6 (4.94) | 87.0 (4.52) | 0.053 | 1114 |  |  |  |
| Sex: |  |  |  |  | 0.496 | 1120 |  |  |  |
| Men | 192 (23.4%) | 2 (14.3%) | 67 (26.3%) | 10 (31.2%) |  |  |  |  |  |
| Women | 627 (76.6%) | 12 (85.7%) | 188 (73.7%) | 22 (68.8%) |  |  |  |  |  |
| Education: |  |  |  |  | 0.356 | 1096 |  |  |  |
| Elementary | 259 (32.3%) | 2 (14.3%) | 76 (30.6%) | 10 (31.2%) |  |  |  |  |  |
| High school | 407 (50.7%) | 10 (71.4%) | 141 (56.9%) | 18 (56.2%) |  |  |  |  |  |
| University | 136 (17.0%) | 2 (14.3%) | 31 (12.5%) | 4 (12.5%) |  |  |  |  |  |
| # chronic diseases | 4.58 (1.86) | 10.8 (2.97) | 7.67 (2.31) | 8.31 (2.72) | <0.001 | 1120 |  |  |  |
| # drugs | 4.50 (2.99) | 9.36 (5.29) | 7.52 (3.47) | 8.47 (4.08) | <0.001 | 1110 |  |  |  |
| Walking speed | 0.58 (0.42) | 0.51 (0.34) | 0.42 (0.37) | 0.49 (0.42) | <0.001 | 1035 |  |  |  |
| MMSE | 25.0 (7.26) | 28.0 (2.09) | 23.9 (8.32) | 27.5 (2.57) | 0.019 | 963 |  |  |  |
|  |  |  |  |  |  |  |  |  |  |
| **3 years** | | | | | | |  |  |  |
|  | **UNSP N=298** | **RESP-CIRCULA & SKIN N=24** | **CARDIORESP & NEURO N=280** | **NEURO-SENS N=38** | **DROPOUT N=102** | **DEATH N=378** | p.overall | N |  |
| Age | 86.5 (4.69) | 84.7 (3.38) | 86.9 (4.61) | 85.9 (4.02) | 86.2 (4.30) | 90.7 (4.97) | <0.001 | 1114 |  |
| Sex: |  |  |  |  |  |  | 0.117 | 1120 |  |
| Men | 61 (20.5%) | 8 (33.3%) | 77 (27.5%) | 14 (36.8%) | 22 (21.6%) | 89 (23.5%) |  |  |  |
| Women | 237 (79.5%) | 16 (66.7%) | 203 (72.5%) | 24 (63.2%) | 80 (78.4%) | 289 (76.5%) |  |  |  |
| Education: |  |  |  |  |  |  | 0.011 | 1096 |  |
| Elementary | 80 (27.2%) | 5 (20.8%) | 79 (28.5%) | 14 (36.8%) | 32 (32.7%) | 137 (37.5%) |  |  |  |
| High school | 149 (50.7%) | 15 (62.5%) | 153 (55.2%) | 19 (50.0%) | 50 (51.0%) | 190 (52.1%) |  |  |  |
| University | 65 (22.1%) | 4 (16.7%) | 45 (16.2%) | 5 (13.2%) | 16 (16.3%) | 38 (10.4%) |  |  |  |
| # chronic diseases | 5.73 (1.75) | 12.2 (3.96) | 9.36 (2.45) | 10.5 (3.43) | . (.) | . (.) | <0.001 | 640 |  |
| # drugs | 3.94 (2.77) | 7.62 (5.68) | 5.93 (3.28) | 6.84 (4.24) | 4.77 (3.17) | 5.95 (3.57) | <0.001 | 1110 |  |
| Walking speed | 0.56 (0.43) | 0.42 (0.40) | 0.43 (0.41) | 0.46 (0.37) | . (.) | . (.) | 0.001 | 627 |  |
| MMSE | 26.8 (4.81) | 28.4 (1.70) | 26.5 (5.09) | 27.8 (1.66) | 27.0 (3.70) | 20.5 (10.1) | <0.001 | 963 |  |
|  |  |  |  |  |  |  |  |  |  |
| **6 years** | | | | | | | | | |
|  | **UNSP N=109** | **RESP-CIRCULA & SKIN N=31** | **CARDIORESP & NEURO N=195** | **NEURO-SENS N=39** | **DROPOUT N=157** | **DEATH N=589** | p.overall | N |  |
| Age | 85.3 (4.27) | 84.0 (3.79) | 86.0 (4.08) | 85.3 (3.78) | 86.2 (4.25) | 89.9 (5.06) | <0.001 | 1114 |  |
| Sex: |  |  |  |  |  |  | 0.037 | 1120 |  |
| Men | 22 (20.2%) | 10 (32.3%) | 49 (25.1%) | 17 (43.6%) | 31 (19.7%) | 142 (24.1%) |  |  |  |
| Women | 87 (79.8%) | 21 (67.7%) | 146 (74.9%) | 22 (56.4%) | 126 (80.3%) | 447 (75.9%) |  |  |  |
| Education: |  |  |  |  |  |  | 0.018 | 1096 |  |
| Elementary | 30 (27.8%) | 8 (25.8%) | 45 (23.6%) | 12 (30.8%) | 49 (32.0%) | 203 (35.4%) |  |  |  |
| High school | 58 (53.7%) | 19 (61.3%) | 102 (53.4%) | 18 (46.2%) | 79 (51.6%) | 300 (52.3%) |  |  |  |
| University | 20 (18.5%) | 4 (12.9%) | 44 (23.0%) | 9 (23.1%) | 25 (16.3%) | 71 (12.4%) |  |  |  |
| # chronic diseases | 6.49 (1.72) | 14.9 (4.21) | 10.3 (2.70) | 11.5 (3.05) | . (.) | . (.) | <0.001 | 374 |  |
| # drugs | 4.75 (2.86) | 9.45 (4.38) | 8.03 (3.66) | 8.55 (4.57) | . (.) | . (.) | <0.001 | 372 |  |
| Walking speed | 0.55 (0.37) | 0.38 (0.35) | 0.38 (0.35) | 0.41 (0.36) | . (.) | . (.) | 0.001 | 368 |  |
| MMSE | 23.6 (7.57) | 24.9 (6.78) | 24.0 (7.11) | 25.8 (3.93) | . (.) | . (.) | 0.388 | 318 |  |
|  |  |  |  |  |  |  |  |  |  |
| **9 years** | | | | | | | | | |
|  | **UNSP N=33** | **RESP-CIRCULA & SKIN N=24** | **CARDIORESP & NEURO N=119** | **NEURO-SENS N=34** | **DROPOUT N=171** | **DEATH N=739** | p.overall | N |  |
| Age | 84.2 (3.02) | 83.9 (3.34) | 85.0 (3.70) | 84.4 (3.49) | 86.2 (4.23) | 89.3 (5.12) | <0.001 | 1114 |  |
| Sex: |  |  |  |  |  |  | 0.134 | 1120 |  |
| Men | 8 (24.2%) | 9 (37.5%) | 25 (21.0%) | 13 (38.2%) | 34 (19.9%) | 182 (24.6%) |  |  |  |
| Women | 25 (75.8%) | 15 (62.5%) | 94 (79.0%) | 21 (61.8%) | 137 (80.1%) | 557 (75.4%) |  |  |  |
| Education: |  |  |  |  |  |  | 0.112 | 1096 |  |
| Elementary | 11 (33.3%) | 5 (20.8%) | 29 (24.4%) | 6 (17.6%) | 51 (30.5%) | 245 (34.1%) |  |  |  |
| High school | 16 (48.5%) | 17 (70.8%) | 66 (55.5%) | 18 (52.9%) | 89 (53.3%) | 370 (51.5%) |  |  |  |
| University | 6 (18.2%) | 2 (8.33%) | 24 (20.2%) | 10 (29.4%) | 27 (16.2%) | 104 (14.5%) |  |  |  |
| # chronic diseases | 7.21 (1.67) | 15.1 (4.26) | 11.4 (2.95) | 13.4 (2.65) | . (.) | . (.) | <0.001 | 210 |  |
| # drugs | 4.58 (2.48) | 7.54 (4.40) | 6.47 (3.07) | 7.26 (3.95) | 8.64 (5.60) | 8.28 (4.26) | <0.001 | 372 |  |
| Walking speed | 0.54 (0.33) | 0.34 (0.30) | 0.38 (0.32) | 0.39 (0.37) | . (.) | . (.) | 0.061 | 209 |  |
| MMSE | 23.9 (8.24) | 27.4 (1.78) | 25.9 (4.77) | 26.7 (3.60) | 26.2 (2.17) | 20.8 (8.78) | <0.001 | 318 |  |
|  |  |  |  |  |  |  |  |  |  |
| **12 years** | | | | | | | | | |
|  | **UNSP N=6** | **RESP-CIRCULA & SKIN N=15** | **CARDIORESP & NEURO N=49** | **NEURO-SENS N=24** | **DROPOUT N=191** | **DEATH N=835** | p.overall | N |  |
| Age | 83.3 (3.57) | 83.6 (2.80) | 83.9 (2.94) | 82.9 (2.52) | 85.9 (4.20) | 88.9 (5.10) | <0.001 | 1114 |  |
| Sex: |  |  |  |  |  |  | 0.370 | 1120 |  |
| Men | 3 (50.0%) | 3 (20.0%) | 10 (20.4%) | 7 (29.2%) | 38 (19.9%) | 210 (25.1%) |  |  |  |
| Women | 3 (50.0%) | 12 (80.0%) | 39 (79.6%) | 17 (70.8%) | 153 (80.1%) | 625 (74.9%) |  |  |  |
| Education: |  |  |  |  |  |  | 0.212 | 1096 |  |
| Elementary | 3 (50.0%) | 3 (20.0%) | 13 (26.5%) | 3 (12.5%) | 57 (30.5%) | 268 (32.9%) |  |  |  |
| High school | 2 (33.3%) | 11 (73.3%) | 27 (55.1%) | 13 (54.2%) | 98 (52.4%) | 425 (52.1%) |  |  |  |
| University | 1 (16.7%) | 1 (6.67%) | 9 (18.4%) | 8 (33.3%) | 32 (17.1%) | 122 (15.0%) |  |  |  |
| # chronic diseases | 6.67 (3.27) | 18.4 (4.60) | 13.1 (3.33) | 15.6 (3.13) | . (.) | . (.) | <0.001 | 94 |  |
| # drugs | 5.00 (5.73) | 11.0 (5.74) | 7.86 (3.64) | 9.00 (4.16) | . (.) | . (.) | 0.019 | 94 |  |
| Walking speed | 0.38 (0.37) | 0.35 (0.27) | 0.38 (0.36) | 0.35 (0.37) | . (.) | . (.) | 0.984 | 92 |  |
| MMSE | 19.8 (11.2) | 22.2 (10.7) | 21.4 (8.50) | 23.8 (7.15) | . (.) | . (.) | 0.727 | 80 |  |

Unspecific (USP); Respiratory-circulatory and skin (RESP-CIRCULA & SKIN); Cardio-respiratory and Neurological (CARDIORESP & NEURO); and Neuro-sensorial (NEURO-SENS). MMSE: Mini Mental State Examination
